# A rabies lesson improves rabies knowledge amongst primary school children in Zomba, Malawi

**DOI:** 10.1371/journal.pntd.0006293

**Published:** 2018-03-09

**Authors:** Jordana L. Burdon Bailey, Luke Gamble, Andrew D. Gibson, Barend M. deC. Bronsvoort, Ian G. Handel, Richard J. Mellanby, Stella Mazeri

**Affiliations:** 1 Mission Rabies, Cranborne, Dorset, United Kingdom; 2 The Roslin Institute and The Royal (Dick) School of Veterinary Studies, Division of Genetics and Genomics, The University of Edinburgh, Easter Bush Veterinary Centre, Roslin, Midlothian, United Kingdom; 3 The Roslin Institute and The Royal (Dick) School of Veterinary Studies, Division of Veterinary Clinical Studies, The University of Edinburgh, Hospital for Small Animals, Easter Bush Veterinary Centre, Roslin, Midlothian, United Kingdom; Wistar Institute, UNITED STATES

## Abstract

Rabies is an important neglected disease, which kills around 59,000 people a year. Over a third of these deaths are in children less than 15 years of age. Almost all human rabies deaths in Africa and Asia are due to bites from infected dogs. Despite the high efficacy of current rabies vaccines, awareness about rabies preventive healthcare is often low in endemic areas. It is therefore common for educational initiatives to be conducted in conjunction with other rabies control activities such as mass dog vaccination, however there are few examples where the efficacy of education activities has been assessed. Here, primary school children in Zomba, Malawi, were given a lesson on rabies biology and preventive healthcare. Subsequently, a mass dog vaccination programme was delivered in the same region. Knowledge and attitudes towards rabies were assessed by a questionnaire before the lesson, immediately after the lesson and 9 weeks later to assess the impact the lesson had on school children’s knowledge and attitudes. This assessment was also undertaken in children who were exposed to the mass dog vaccination programme but did not receive the lesson. Knowledge of rabies and how to be safe around dogs increased following the lesson (both p<0.001), and knowledge remained higher than baseline 9 weeks after the lesson (both p<0.001). Knowledge of rabies and how to be safe around dogs was greater amongst school children who had received the lesson compared to school children who had not received the lesson, but had been exposed to a rabies vaccination campaign in their community (both p<0.001) indicating that the lesson itself was critical in improving knowledge. In summary, we have shown that a short, focused classroom-based lesson on rabies can improve short and medium-term rabies knowledge and attitudes of Malawian schoolchildren.

## Author summary

Rabies is a fatal disease that claims the lives of approximately 59,000 people every year. Children under the age of 15 make up 40% of all human rabies deaths yet this is preventable through a combination of vaccinating dogs against rabies and education. Numerous studies have shown that people in rabies endemic areas lack sufficient knowledge about rabies, and there are many misconceptions about its treatment and prevention. Whilst many organisations run vaccination and education campaigns, few have assessed their impact on rabies knowledge, attitudes or practices (KAP). Fewer still have assessed the impact on children. This study investigated the impact of a rabies lesson on school children’s knowledge and attitudes about rabies in conjunction with a rabies vaccination campaign in Zomba, Malawi. We found that a rabies lesson improved school children’s knowledge about rabies and how to be safe around dogs. We observed that knowledge remained higher several weeks later. Knowledge about both canine rabies and bite prevention was greater amongst school children who had received the lesson compared to school children who had not received the lesson, but had been exposed to a rabies vaccination campaign in their community. This indicates that the lesson itself was critical in improving knowledge.

## Introduction

Of the estimated 59,000 people who die from rabies annually [1], the vast majority result from a bite from a rabid dog. Children are at greater risk of suffering dog bites than adults [2,3] and as a result approximately 40% of all human rabies deaths occur in children aged under 15 years old [4,5]. Elimination of the rabies virus can be achieved through annual vaccination of 70% of the dog population and human exposures to rabies virus will continue to occur until elimination has been achieved. Prompt post-exposure treatment is effective at preventing rabies, however incomplete adherence to recommended protocols has resulted in many deaths.

School based rabies education is an efficient way of reaching large numbers of children. Lessons containing simple messages can improve rabies prevention through appropriate behaviour, such as immediately washing bite wounds and seeking post-exposure vaccination.

Whilst many governments and NGOs advocate the integration of education components in rabies elimination programmes [6], few have published studies documenting the effectiveness of their interventions [7–9].

Knowledge, attitudes and practices (KAP) studies can be used to assess how effective education initiatives are by comparing responses prior to, and after, an intervention. Studies have shown that knowledge of rabies and rabies prevention can vary greatly across rabies endemic countries. For example, in one rabies KAP study in Tanzania only 5% of those interviewed knew of the importance to thoroughly wash dog bite wounds [10]; over 35% of respondents in Ethiopia did not know the symptoms of rabies in people [11]; and in Cambodia only 48% of people knew that vaccination could protect dogs from rabies [4]. A lack of understanding about the risk of rabies and preventive measures reduces the perceived need for control measures. It also reduces engagement with elimination efforts and the likelihood of taking appropriate action to prevent rabies in the event of exposure. Most rabies KAP studies have focused on adult populations [4,10–20] despite the disproportionally high incidence of rabies in children [8]. Only two studies have evaluated the efficacy of lessons to improve rabies KAP in children; Kanda *et al*. in Sri Lanka [9] and Dzikwi *et al*. in Nigeria [21]. Furthermore, studies assessing longer term knowledge retention after a short lesson and comparison with control populations exposed to mass dog vaccination campaigns alone are lacking. The current study was therefore conducted to investigate the immediate and medium-term impact of short lessons on primary school children’s understanding of rabies prevention in Zomba City, Malawi.

## Methods

### Study site

**Fig 1:**
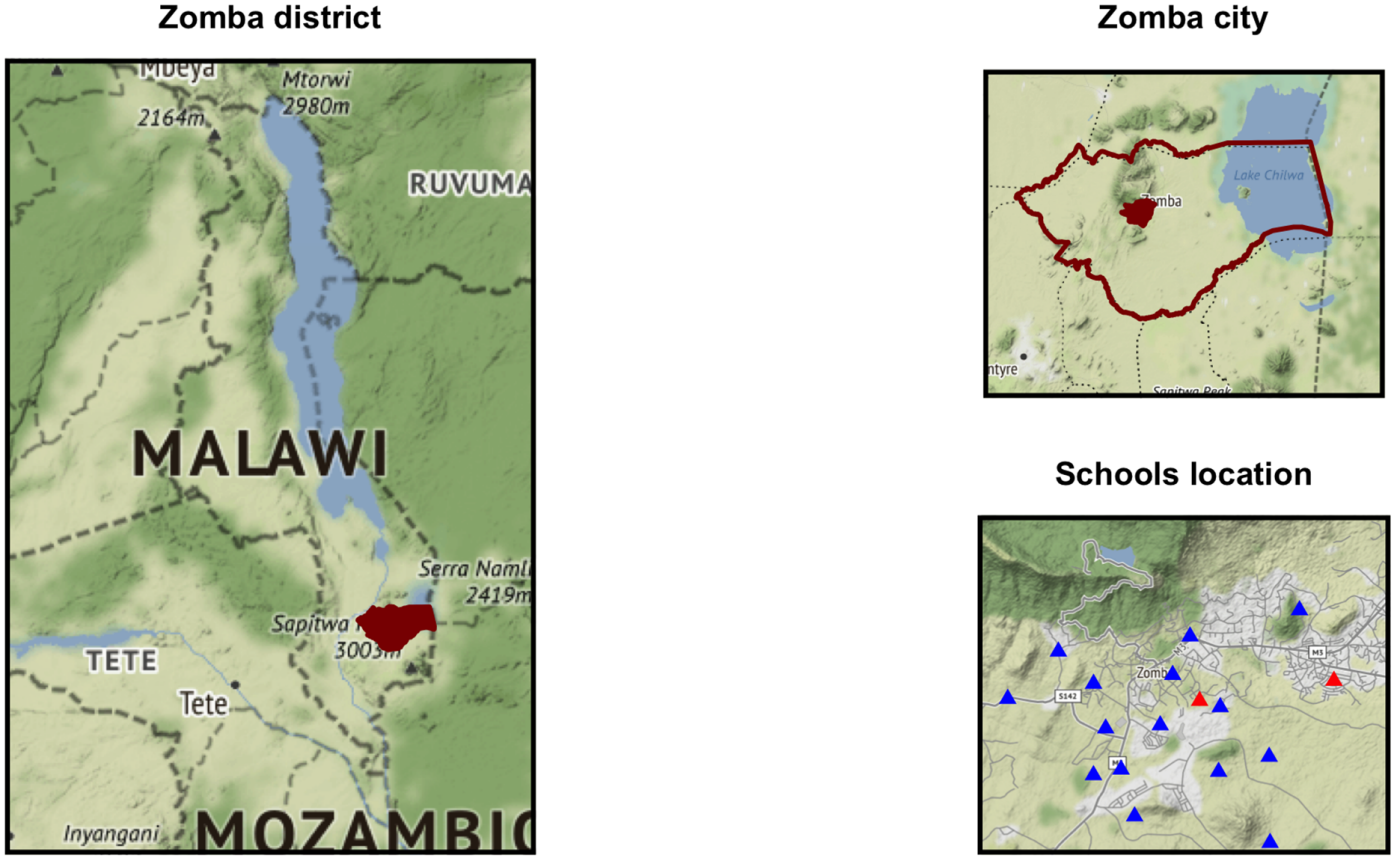
Map of Zomba, Southern Malawi, with primary schools identified. Blue triangles identify the location of primary schools that received a rabies lesson. Red triangles identify the location of control schools. Map was plotted using R package *ggmap* [23] and the map tiles were sourced from Stamen Design (using data by OpenStreetMap), which are freely available under CC BY 3.0 license.

Zomba city, a rabies education naïve area in Southern Malawi, was chosen as the study site (Fig 1). Zomba City, located in Zomba District of southern Malawi is the fourth largest city in Malawi, with a human population of 114,000 (based on 3.2% annual growth rate since the 2008 census [22]). Mission Rabies is an international NGO working to establish effective rabies control activities in Malawi, including mass dog vaccination, community rabies education and enhanced canine rabies surveillance initiatives. Mass dog vaccination and education campaigns were conducted in Blantyre city in May 2015 and May 2016. No previous education or vaccination activities had taken place in Zomba city prior to this study. Local reports of high rates of canine and human rabies and an absence of rabies vaccination or education activities prompted requests from local authorities for expansion of Mission Rabies activities to the Zomba region. This study was undertaken during the initial stages of work in Zomba.

### Education programme

The 17 public primary schools in Zomba City had a total of 25,824 registered school children in 2016. All schools were included in the study. Fifteen received rabies education classes shortly before a mass dog vaccination campaign in the region. Two control schools did not receive education classes prior to the vaccination campaign. The control schools were closed during the period before vaccination so were not available to conduct the rabies education classes before the campaign. To investigate whether the choice of control introduced a bias, being a convenient sample, demographics of children in the intervention schools and the controls schools were compared.

Lessons were delivered in the national language, Chichewa, by trained Malawian Mission Rabies education officers to children in the seventh school year (standard 7). The interactive lessons lasted for approximately 45 minutes and included school children participation, requiring volunteers to join in short demonstrations and question-answer sessions. Learning points included which animals can transmit rabies, rabies symptoms and prevention, and safety around dogs. Education officers received four days of training in how to deliver a standardised lesson before commencing the study. The Mission Rabies school education campaign teaches all year groups, with lessons ranging from 20 minutes for younger years to 60 minutes for older year groups. A single year group was included in this study to standardise the type of lesson delivered.

### Education programme evaluation

The education programme was evaluated using self-administered paper questionnaires. Rabies lessons and initial questionnaires were conducted between 11^th^ and 17^th^ July 2016. In the 15 schools where rabies lessons were given, the same standardised questionnaire was completed by school children at three time-points; a “pre” questionnaire prior to receiving the lesson, a “post” questionnaire by the same children immediately following the lesson and a “retention” questionnaire, in the same class at the same schools 7.5 to 10.5 weeks later. The pre-questionnaire was used to assess children’s baseline knowledge and attitudes; the post-questionnaire assessed instant impact of the lesson on school children’s knowledge and attitudes; and the retention questionnaire assessed longer-term learning.

The control group completed the same questionnaire only once (“control” questionnaire), after the vaccination campaign that took place between 6^th^ and 17^th^ August 2016. This approach allowed us to assess the impact of the rabies lesson itself rather than just the exposure to the wider dog rabies vaccination programme. Retention and control questionnaires were completed between 8^th^ and 29^th^ September 2016.

### Questionnaire

The initial draft of the questionnaire was designed using input from NGO staff and publications conducting similar questionnaires directed at adults, followed by a process of informal feedback and refinement through application in two schools in Blantyre city. The questionnaire was written in English and translated to Chichewa. It was independently back translated to English for comparison with the original version to ensure question integrity was maintained.

The questionnaire consisted of four sections. Section A identified demographic information. Section B assessed dog ownership practices and understanding of animal welfare (to be reported outside of this study). Section C contained questions about rabies. Section D asked about prior rabies education and dog vaccination history. This section was used by the NGO and did not form part of this study. A copy of the questionnaire can be found in S1 Appendix.

### Participant selection

To minimise age related bias the questionnaire was given to standard 7 school children only, as described above. Sample size was determined using a sample size calculator [24] with the following parameters: 95% confidence level; 5% margin of error; and a response distribution of 50%. These parameters were chosen to give the most conservative sample size. The target population contained 2,844 standard 7 school children registered with the education department in Zomba, therefore a sample size of 339 was required. This was the equivalent of 22.6 school children per school for the educated group and 169 per school for the control group. To account for incomplete questionnaires and varying school attendance this was increased to 30 per school for the educated group and 190 per school for the control group.

Even though each year group has an average of 169 registered students, which would lend itself well to systematic random sampling of every fifth student from the class register, attendance was much lower. For this reason, the teacher selected 30 children to take part, or as many as were present in the class where there were less than 30 school children. The teacher was instructed to select school children at random and not to choose by ability. All school children had the opportunity to decline taking part at each stage of the study.

### Anonymity

Anonymity was maintained throughout the study by allocating each participant a unique identification code (UIC) consisting of a school code followed by a sequential number. The UIC was entered on the questionnaire, a consent letter for school children’s parents/guardians and on the data entry smartphone application. Less than half of the school children could be reliably matched between the pre and retention questionnaire due to school children absence at the time of the retention questionnaire or because school children forgot their UIC. Where there were fewer school children present for the retention than for the pre/post questionnaire, additional school children were invited to take the questionnaire with the provision that they had been present for the rabies lesson. The UIC was adapted to take this into consideration.

### Data collection

All questionnaires were presented to children on paper. Results from the questionnaires were entered into digital versions of the questionnaire created in a smartphone application (the Mission Rabies App) that allows remote data collection through smartphones [25]. Where possible, data were entered through single- and multi-select options to minimise transcription error [26]. Data were uploaded to a Microsoft SQL database on a secure cloud based server, from which it could be downloaded remotely via a password protected website as a csv file, which was imported into Excel 2013 (Microsoft Inc., Redmond, WA) and R Studio [27] for analysis.

### Statistical analysis

To allow statistical analysis numerical scores were allocated to answers based on accuracy of response for questions which had correct or incorrect answers. A completely correct answer scored 2, a mostly correct answer 1, a missing or wrong answer 0 and an incorrect answer -1. For example, the latter includes answers that could have deleterious consequences for the child or an animal, or if an incorrect species susceptible to rabies was identified. Scores of 0 were given to answers that are not correct but would not have deleterious consequences. For example, the risk of getting rabies from milk is theoretical but people are not encouraged to drink milk from a rabid animal [28,29]. Therefore, responses that milk could transmit rabies were given a score of 0, as whilst it is wrong it is not harmful. Scores allocated to each question response can be found in S1 Table. When questions allowed for multiple answers the score was the sum of all answers selected. Questions were grouped into categories that assessed knowledge and attitudes towards rabies and safety around dogs (S2 Table).

Data were analysed using the R statistical software version 3.3.2 [30] with paired t-tests comparing matched pre and post questionnaire responses [31]. Results from 13 pre-questionnaires could not be matched to post questionnaires and these data was excluded when performing paired t-tests between these questionnaires. Two tailed two sample t-tests [31] were used to compare data that could not be matched: pre to retention, pre to control and control to retention scores. To deal with the issue of multiple testing, the threshold cut-off for significance was adjusted to a p-value < 0.003 based on the Bonferroni correction [31]. Mixed effects multiple linear regression [31] was used to determine the effect of demographics on baseline questionnaire scores. Children who had not replied to any of the questions considered in the model were removed from the regression analysis. Children’s age, gender, religion and dog ownership status were considered in the model as fixed effects. The school each student studied at was introduced in the model as a random effect. Variables selection was carried out using manual backward elimination and variables retained in the final regression model were chosen based on their effect on the Akaike information criterion (AIC).

### Ethics

The study and questionnaire were approved by the University of Edinburgh’s Human (Research) Ethical Review Committee (HERC). In Malawi, we obtained permission from the Department of Education. Signed consent was granted by head teachers of all schools prior to commencing the study. Consent letters were given to school children to give to their parents/guardians with clear instructions on how to remove their child’s data from the study if desired.

## Results

### School children demographics

**Fig 2:**
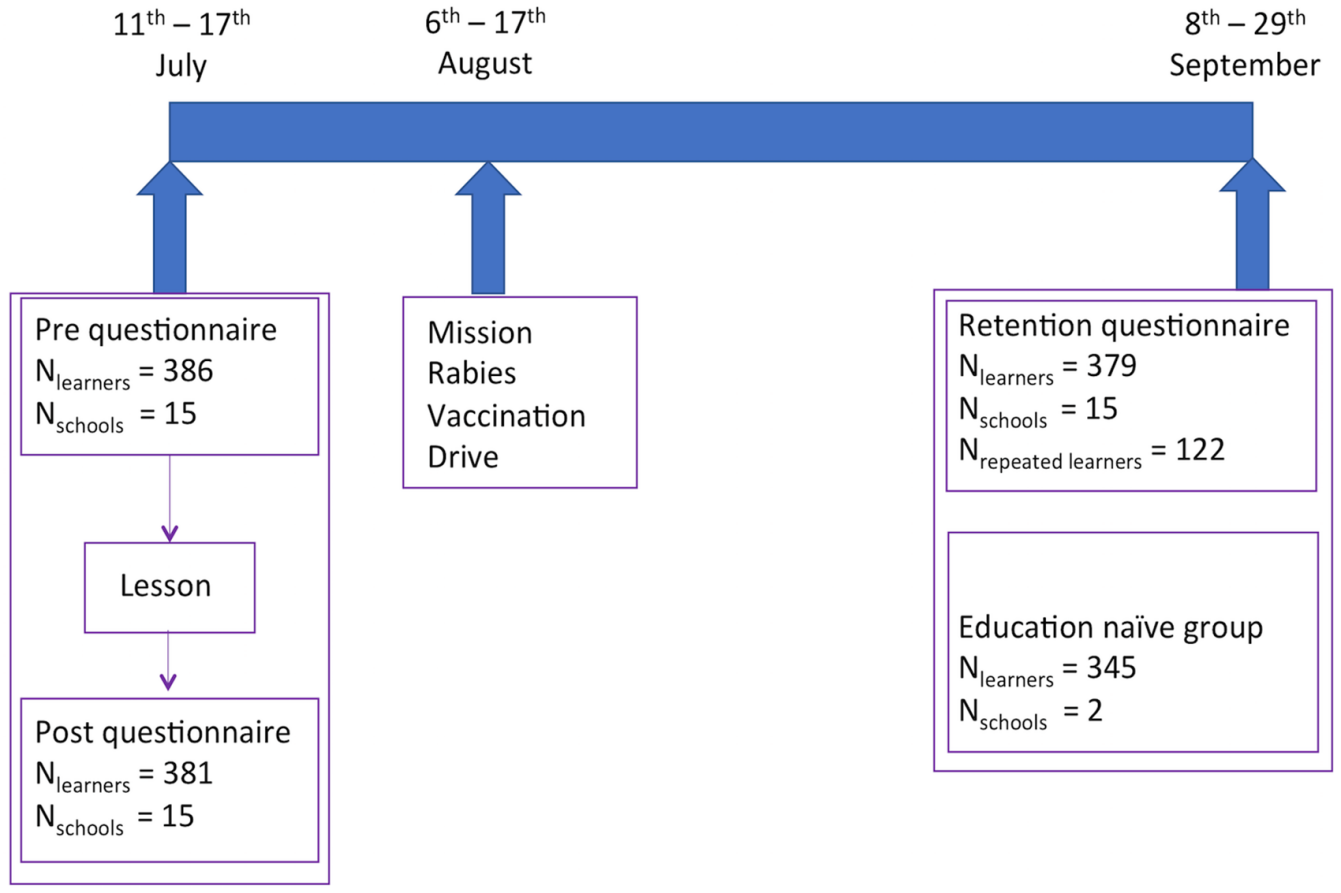
Diagram illustrating working schedule for questionnaire delivery in relation to rabies lessons and rabies vaccination campaign. The number of school children completing questionnaires at each stage are indicated. N_repeated school children_ are the number of school children from the pre-questionnaire who could be accurately identified for the retention questionnaire.

The number of school children who completed the questionnaire at each stage is shown in Fig 2. Only 122 out of 386 school children who completed the pre-questionnaire and could remember their UIC completed the retention questionnaire. School children who had been present for the lesson but did not complete the questionnaire and those who could not remember their UIC comprised the remainder of school children completing the retention questionnaire (Fig 2). S1 Fig shows the distribution of missing data in the questionnaire responses.

The mean age of school children was 13 years old with a range between eight and 15 years old for pre, post and control groups, and nine and 15 years old for retention groups. Though approximately equal, slightly more females than males completed the questionnaire.

Approximately 50% of the educated school children were Catholic, with other Christian denominations accounting for at least 30%. This differed in the control group where more school children belonged to other Christian denominations followed by Catholicism. Across all groups between nine and 15% of school children were Muslim. Across all questionnaires the majority of school children either owned or had contact with dogs (pre 78.8%, n = 304; post 79.3%, n = 302; retention 83.6%, n = 317; control 82.3%, n = 284).

The main reason for dog ownership was guarding (pre 86.1%, n = 242; post 86.3%, n = 276; retention 81.8%, n = 233; control 86.1%, n = 223). Most dogs were kept tied up or in a cage outside (combined results: pre 76.2%, n = 294; post 74.5%, n = 284; retention 68.9%, n = 261; control 58.6%, n = 202).

S2 Fig shows graphical comparisons of means/proportions and confidence intervals of demographic characteristics between intervention (educated school children) and control (school children not exposed to the rabies lesson).

### Baseline knowledge and attitudes of school children

Pre-questionnaire results were used to determine baseline knowledge and attitudes. Overall, this was assessed by pooling the scores for all questions assessing knowledge or attitudes. Accounting for school differences as a random effect, the mixed effects multiple linear regression model showed that male students had higher baseline scores. Similarly, when compared to Catholic students, students who said they did not belong to a religious group had higher scores. School children that did not own or have contact with dogs had lower overall baseline scores when compared to those who owned dogs. Results of the regression model are presented in Table 1. The variables selection process used is explained in Table 2.

**Table 1:** Linear regression model of factors associated with baseline knowledge.

Factor	Estimate	95% CI	p-value
**Age**	0.52	-0.14–1.18	0.122
**Gender**			
Female	Reference level		
Male	1.86	0.04–3.68	0.046
**Religion**			
Catholic	Reference level		
Christian other	1.28	-0.55–3.11	0.172
Muslim	-1.29	-3.82–1.24	0.319
None	5.92	0.32–11.53	0.039
Other	2.36	-5.51–10.23	0.557
**Dog ownership**			
Own	Reference level		
Contact	-0.36	-2.22–1.49	0.701
None	-2.33	-4.64-0.01	0.049

The table shows the results of a linear regression model showing the effects of each variable on children’s baseline knowledge.

**Table 2:** Variable selection for regression model.

Model	Difference in AIC
All variables included*	0
All except gender	+3.68
All except dog ownership	+3.88
All except religion	+12.69

Table shows the variables selection process. Using manual backward elimination each variable was removed from the model and the variable was kept in the model if AIC increased. In this situation, this was the case for all variables hence all variables were kept in the final model.

*Children’s age, gender, religion and dog ownership.

Safety around dogs was assessed by asking school children how to behave around dogs to avoid being bitten. Of all responses, 86.6% identified an appropriate behaviour (stand still, be calm, cover face and play with friendly dogs) (S3 Fig).

School children achieved a mean score of 19.23 (sd 7.89) out of 71 for rabies knowledge. Seven out of the eight questions assessing rabies knowledge allowed multiple responses, yet between 43.0% and 72.5% of school children gave single answers to these questions. Most school children knew that people can get rabies (86.5%, n = 334). 91.0% (n = 628) of responses for the question ‘Which animals can get rabies?’ were correct with 49.0% (n = 338) of school children selecting dogs, whilst only 83.3% (n = 685) of responses were correct for ‘Which animals can give rabies to people?’. Being bitten was the most commonly selected option for how people can get rabies with 59.4% (n = 318) of responses. Many school children could identify symptoms of rabies (95.6%, n = 723 correct responses). Of the responses for ‘What do you do if bitten by a dog?’ 24.2% (n = 172) of responses were correct and 68.4% (n = 486) were completely correct. Over half of the responses (54.0% n = 249) given to the question asking children to identify ways to prevent dogs from getting rabies were that a dog should be vaccinated annually. School children also identified that vaccinating dogs and people would prevent people from getting rabies (32.2% n = 170 and 39.2%, n = 207 responses respectively). S3 Table details rabies knowledge responses across all questionnaires. Most school children believed that rabies was serious and that dogs should be vaccinated giving an overall rabies attitude score of 3.81, out of 4.

### Assessing the impact of the lesson

**Fig 3:**
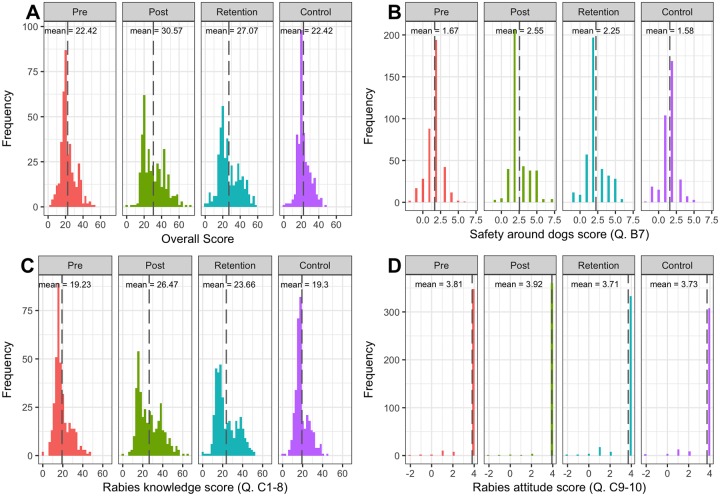
Plots showing scores for the different categories examined for each questionnaire type with the mean score for each indicated. a) Overall score b) Safety around dogs c) Rabies Knowledge d) Rabies Attitudes.

Overall score results, as well as scores for each category are presented in Fig 3. Furthermore, results of t-test comparisons are shown in Table 3. Results for overall score, safety around dogs and rabies knowledge all demonstrated a significant improvement in score immediately after the lesson. This reduced over time but remained significantly greater than baseline at the retention questionnaire. Control scores were significantly different to retention scores but not significantly different to pre-questionnaire scores.

**Table 3:** Mean, 95% confidence interval and t-tests for questions assessing knowledge and attitudes towards rabies.

	Mean 1	Mean 2	95% CI*	p-value
**Safety around dogs**				
Post -v- Pre**	2.56	1.67	0.71–1.022	<0.0005
Retention -v- Pre	2.25	1.67	0.401–0.747	<0.005
Control -v- Pre	1.58	1.67	-0.247–0.064	0.025
Retention -v- Control	2.25	1.58	0.493–0.839	<0.005
**Rabies Knowledge**				
Post -v- Pre**	26.38	19.23	6.245–8.051	<0.0005
Retention -v- Pre	23.66	19.23	3.069–5.800	<0.0005
Control -v- Pre	19.3	19.23	-1.017–1.158	0.899
Retention -v- Control	23.66	19.3	3.037–5.69	<0.0005
**Rabies Attitude**				
Post -v- Pre**	3.91	3.81	0.018–0.177	0.016
Retention -v- Pre	3.71	3.81	-0.234–0.022	0.106
Control -v- Pre	3.73	3.81	-0.210–0.047	0.215
Retention -v- Control	3.71	3.73	-0.166–0.118	0.738
**Overall score**				
Post -v- Pre**	37.7	28.68	7.973–10.107	<0.0005
Retention -v- Pre	33.91	28.68	3.572–6.880	<0.0005
Control -v- Pre	29.28	28.68	-0.740–1.940	0.38
Retention -v- Control	33.91	29.28	2.992–6.261	<0.0005

Mean 1 = mean of first questionnaire type mentioned in corresponding row. Mean 2 = mean of second questionnaire type mentioned in corresponding row. The Bonferroni adjusted threshold cut-off value for a type I error of 5% was P < 0.003 to account for multiple testing.

*95% Confidence Interval of the difference between the means,

**paired t-test.

Some question responses illustrated an improvement in knowledge more than others. For example, 32% of children identified that they should inform an adult if they were bitten by a dog after the lesson compared to 17% before the lesson. They also knew to clean the wound for 15 minutes (proportion of responses per questionnaire: pre 1.8% n = 13, post 9.9% n = 81, retention 8.8% n = 65) and apply antiseptic (proportion of responses per questionnaire: pre 4.5% n = 32, post 10.3% n = 84, retention 9.3% n = 69). This knowledge diminished over time but remained greater than baseline levels 9 weeks after the lesson. Conversely school children failed to identify that they should go for 5 vaccines and go to the hospital (S4 Fig).

Following the lesson there was an increase in the number of animals that school children identified could get rabies. The number of responses increased for all mammal species, though the greatest difference was in cats, bats and monkeys. This knowledge waned overtime though remained greater than baseline (for all mammal species other than dogs) and was greater than control groups (except for mongoose). Equally, there was a very similar increase in the number of animals school children identified that could transmit rabies to people. Again, the number of responses increased for all mammals with the greatest increase for cats, bats and monkeys. Children’s responses to the question assessing how rabies can be transmitted to people followed a similar pattern with an increase in correct responses that diminished over time but remained greater than baseline and the control group for all but one of the correct answers. There was an increase in response rate to saliva and scratches, and to a lesser extent licking wounds, after the lesson. However, this was coupled with a reduction in children’s responses indicating that bites can transmit rabies to people. There were no significant changes between school children groups or questionnaires for attitudes towards rabies (Table 3) as most school children had initially shown strong attitudes. Similarly, over 80% of responses to all questionnaire types indicated that children knew that people could get rabies.

## Discussion

This study examined the ability of a rabies school lesson to enhance both immediate and medium-term rabies knowledge and attitudes in primary school children. To the authors’ knowledge, this is the first study to evaluate medium-term rabies knowledge retention post intervention in children. Additionally, assessment of rabies understanding gained from a lesson in comparison to that generated by high-profile dog rabies vaccination campaigns alone has not been previously reported. We have shown that a short rabies lesson can significantly improve knowledge about rabies biology and preventive healthcare and that this information is largely retained two months later. Children receiving rabies lessons scored better than children who had only been exposed to the vaccination campaign.

For many people in Malawi and other sub-Saharan countries there remain many barriers to accessing appropriate post exposure prophylaxis following a dog bite. These include geographic and economic constraints for individual bite victims. This is exacerbated by frequent shortages of human rabies vaccine at major hospitals and rabies immunoglobulin, for example the latter is not available in Malawi. Until improvements are made in access to effective vaccines, prompt and thorough local wound treatment may be the only opportunity for bite victims to reduce their risk of rabies. Washing bite wounds for a period of at least 15 minutes with water and soap, detergent, povidine iodine or other virucidals can significantly reduce the risk of contracting rabies from an infectious animal [32,33]. This study showed that both before and after the lesson, more children answered that it was necessary to get vaccinated than to wash the wound, highlighting the need to raise common knowledge of this potentially lifesaving step. Hampson *et al*. also reported the lack of awareness of prompt bite wound washing in Tanzania [34]. In the current study, there was a fivefold increase in the proportion of children answering to wash bite wounds for 15 minutes, from 2% to 10% following the lesson. This accompanied a marked increase in the proportion of children reporting the importance of telling an adult from 17% to 32%. Although it is hoped that increasing the likelihood of children reporting bite wounds to adults improves their chances of receiving appropriate PEP, this may not be the case. Despite the statistically significant increases in correct responses, the proportions of students giving completely correct responses remained lower than ideal. The lesson should be reviewed aiming to achieve even greater improvement in children’s knowledge, especially in crucial issues such as the proportion of children knowing that dog bite wounds must be thoroughly washed. This might be achieved through greater emphasis on critical aspects of the lesson, repeating education programmes more often and combining them with other interventions. Community education activities targeting adults take place in conjunction with the school education campaign. Further investigation is warranted to assess whether there is a change in knowledge and practices among the adult population. Given the risk of other water borne diseases such as schistosomiasis, it may be beneficial to incorporate simple messages about the importance of using clean water.

There was no significant change after the lesson in the proportion of children reporting the need for five post-exposure vaccinations. This may be because they selected other behaviours such as telling an adult. Other factors such as: the cost of travel; distance to vaccine distribution points; physician recommendations; and availability of vaccine, are more likely to influence a child’s likelihood of completing a full course of rabies vaccinations regardless of their knowledge on how many doses are needed [34].

Demographic factors associated with baseline rabies knowledge and attitude scores were investigated using a mixed effects multiple linear regression model. Our results showed that male students had higher baseline scores. Additionally, school children that did not own or have contact with dogs had lower overall baseline scores when compared to those who owned dogs. During our experience in the field, we observed that dogs are more often brought to the vaccination clinics by boys rather than girls. Additionally, several authors have suggested that children that have experience with animals have greater knowledge about the welfare needs of animals [35,36]. It is likely that boys, which have more interaction with dogs and are more likely to take up responsibilities relevant to maintaining the health of dogs, might result in them knowing more about dog welfare, diseases and risks relating to interacting with them. Lastly, religion was included in the model as a potential explanatory variable as it was hypothesised that Muslim children, who do not traditionally keep dogs [37,38] and may therefore know less about dog welfare needs, would have lower baseline knowledge. Nevertheless, the model showed no such difference. On the other hand, students who said they did not belong to a religious group had higher knowledge scores compared to Catholic students.

A novel component of this study was the assessment of both the immediate and mid-term (after about 2 months) impact of a rabies lesson on knowledge and attitudes. Dzwiki *et al*. tested effects of a lesson and educational leaflets 2 weeks after the intervention [21], whilst Matibag *et al*. reassessed KAP 4 weeks after distributing leaflets and acknowledged the need to test longer term KAP [7]. Assessing mid to long term knowledge retention has important implications to determine if educational interventions warrant repetition and at what frequency. Whilst there was a slight drop in overall knowledge and attitude scores 9 weeks post lesson, the overall score remained significantly higher than at baseline. The lesson given by the NGO in this study is repeated annually and it would be interesting to assess rabies knowledge and attitude scores at the next visit to determine level of retention after one year and the effects of a repeat lesson.

A child’s risk of contracting rabies may be reduced if they are better able to recognise signs of aggression in dogs and know what they can do to avoid being bitten by aggressive dogs. This study found that children scored better on questions about how to stay safe around dogs after the lesson. The use of roleplay and theatre was a low cost and engaging way to convey aspects of dog behavior without the need for electricity or video equipment as has been described in other studies (19). Additional improvements to knowledge that may reduce a child’s risk of rabies included understanding which animals can transmit the disease and that rabies is transmitted in saliva.

Queen Elizabeth Central Hospital in Blantyre, Malawi, documented the highest number of child rabies deaths from any African hospital [39], making the findings of this study more significant. It is anticipated that improving child rabies knowledge and attitudes will result in reduced child rabies deaths. The finding that rabies knowledge was significantly greater in children who received the lesson compared to children who had only experienced a mass dog vaccination campaign, highlights the benefits of conducting school focused education activities on rabies. In the authors’ experience, children often play a positive role in mass dog vaccination campaigns by bringing dogs for vaccination and increasing vaccination coverage. Therefore, timing school education initiatives shortly before mass vaccination campaigns could have the dual benefit of increasing turnout to vaccination camps and increasing their chances of seeking appropriate PEP in the event of exposure.

This study had some limitations. Due to low attendance of children in each classroom, systematic random sampling was impossible to employ and teachers were asked to choose students to take part in the questionnaire. To overcome the possible bias that can arise from this it was emphasised that the selection should not be based on student’s ability. Additionally, it was not possible to ensure that the school children who completed the pre/post questionnaire also completed the retention questionnaire. This prevented direct comparisons between individuals, however it was still possible to compare results as a population. Despite this the results emphasised the efficacy of a rabies lesson among school children in urban Malawi. Further work is needed to investigate whether the findings of this study can be repeated in rural populations. Furthermore, controls for this study were chosen conveniently. To investigate potential bias introduced by this, demographics of intervention and control groups were compared. The only difference found regarding the demographics between the groups, was the proportion of Catholic students compared to Christians from other denominations. Based on our regression analysis, this is not expected to have an effect on baseline rabies knowledge, so it is unlikely to introduce bias to our work. One important result of this study is that we have been able to show significant knowledge retention in our intervention group. Despite that, the lack of a control group where students would be exposed to the educational campaign, but not the vaccination campaign makes it difficult to distinguish whether retention was due to the rabies lesson alone or was enhanced by exposure to the vaccination campaign. Finally, the change in risk of rabies or exposure to dog bites in children following the lesson could not be evaluated in the current study. Therefore, future investigation into changes in risk reduction behavior following the lesson is warranted.

Although the described education activities took place under the consent and support of the Department of Education and it is delivered by Malawian Education officers, there remain challenges in scaling this initiative to larger, particularly rural areas in a cost-effective way. The use of external education officers who systematically tour schools in a region, delivering specific health care messages has benefits in bringing up-to-date information and teaching methods. However, the duration of each class and benefit delivered needs careful consideration. Future work should explore how lessons such as those described here could be integrated into the national curriculum and effectively disseminated to teachers to give in remote areas across the country. Further study could focus on the effect of lessons delivered by remotely trained Malawian school teachers as a part of a framework that could be implemented nationally.

### Conclusion

This study assessed the impact of a rabies lesson on an education naïve population and demonstrated that this is an effective way to improve knowledge in primary school children in an urban setting. Some aspects of the lesson were more effective than others in teaching children about rabies and its prevention. Knowledge remained greater than baseline suggesting the lesson allowed mid-term learning, though research to determine long-term knowledge retention is warranted. The educational component of this study took place alongside a rabies vaccination campaign and it was demonstrated that the vaccination campaign did not alter children’s knowledge about rabies or how to be safe around dogs. Rabies attitudes did not alter after the lesson but this was because children had already identified that rabies was serious prior to the lesson. The lesson format presented in this study was effective at teaching school children about rabies and its prevention using few resources and training. This study was conducted in the city of Zomba. We therefore believe that this lesson could be successfully used throughout urban Malawi providing children with effective techniques to reduce child mortality from this fatal disease. Future research is needed to assess the efficacy of this lesson in the rural setting and whether the increase in knowledge correlates to reduced risk of rabies.

## Supporting information

S1 Appendix Questionnaire. (DOCX)

S1 Fig Distribution of missing data. (PDF)

S2 Fig Comparison of learners’ demographics between intervention and control group. (PDF)

S3 Fig Responses given to the question ‘how to behave around dogs to prevent being bitten’. (PDF)

S4 Fig Responses to the question “What to do if bitten by a dog” across questionnaires. (PDF)

S1 Table Scoring system for questionnaire responses. (DOCX)

S2 Table Question categorization. (DOCX)

S3 Table Responses to rabies knowledge questions as a total number of children and as a percentage for each questionnaire category. (DOCX)

S1 Checklist STROBE checklist. (DOCX)

## References

[1] Hampson K, Coudeville L, Lembo T, Sambo M, Kieffer A, Attlan M, et al Estimating the Global Burden of Endemic Canine Rabies. 2015;0: 1–20. doi: 10.1371/journal.pntd.0003709 25881058 10.1371/journal.pntd.0003709 PMC4400070

[2] Griego RD, Rosen T, Orengo IF, Wolf JE. Dog, cat, and human bites: A review. J Am Acad Dermatol. 1995;33: 1019–1029. 7490347 10.1016/0190-9622(95)90296-1

[3] Schalamon J, Ainoedhofer H, Singer G, Petnehazy T, Mayr J, Kiss K, et al Analysis of Dog Bites in Children Who Are Younger Than 17 Years. Pediatrics. 2006;117: e374–e379. doi: 10.1542/peds.2005-1451 16510617 10.1542/peds.2005-1451

[4] Lunney M, Fèvre SJS, Stiles E, Ly S, San S, Vong S. Knowledge, attitudes and practices of rabies prevention and dog bite injuries in urban and peri-urban provinces in Cambodia, 2009. Int Health. Royal Society of Tropical Medicine and Hygiene; 2012;4: 4–9. doi: 10.1016/j.inhe.2011.12.001 24030875 10.1016/j.inhe.2011.12.001

[5] WHO. Expert Consultation on Rabies. Second report. 2013. 24069724

[6] Global Alliance for Rabies Control. In: https://rabiesalliance.org/resources/teaching-children/. 2017.

[7] Matibag GC, Ohbayashi Y, Kanda K, Yamashina H, Bandula Kumara W, Gamini Perera IN, et al A pilot study on the usefulness of information and education campaign materials in enhancing the knowledge, attitude and practice on rabies in rural Sri Lanka. J Infect Dev Ctries. 2009;3: 55–64. 19749450 10.3855/jidc.106

[8] Dzikwi AA, Ibrahim AS, Umoh JU. Knowledge and Practice about Rabies among Children Receiving Formal and Informal Education in Samaru, Zaria, Nigeria. Glob J Health Sci. 2012;4: 132–139. 10.5539/gjhs.v4n5p132 PMC4776965 22980386

[9] Kanda K, Obayashi Y, Jayasinghe A, Gunawardena GSPDS, Delpitiya NY, Priyadarshani N, et al Outcomes of a school-based intervention on rabies prevention among school children in rural Sri Lanka. Int Health. 2014;7: 348–353. doi: 10.1093/inthealth/ihu098 25549632 10.1093/inthealth/ihu098

[10] Sambo M, Lembo T, Cleaveland S, Ferguson HM, Sikana L, Simon C, et al Knowledge, Attitudes and Practices KAP) about Rabies Prevention and Control: A Community Survey in Tanzania. 2014;8 doi: 10.1371/journal.pntd.0003310 10.1371/journal.pntd.0003310 PMC4256472 25473834

[11] Tschopp R, Bekele S, Aseffa A. Dog Demography, Animal Bite Management and Rabies Knowledge-Attitude and Practices in the Awash Basin, Eastern Ethiopia. PLoS Negl Trop Dis. 2016; 1–14. doi: 10.1371/journal.pntd.0004471 26900855 10.1371/journal.pntd.0004471 PMC4768771

[12] Jamile L, Emanuel M, Fernandes B. Rabies: Knowledge and Practices Regarding Rabies in Rural Communities of the Brazilian Amazon Basin. PLoS Negl Trop Dis. 2016;10: e0004474 doi: 10.1371/journal.pntd.0004474 26927503 10.1371/journal.pntd.0004474 PMC4771201

[13] Opaleye OO, Adesiji YO, Olowe OA, Fagbami AH. Rabies and antirabies immunization in South Western Nigeria: knowledge, attitude and practice. 2006;452: 116–117. 10.1258/004947506776593305 16611452

[14] Mai L, Dung L, Tho N, Quyet N, Than P, Mai N, et al COMMUNITY KNOWLEDGE, ATTITUDES, AND PRACTICES TOWARD RABIES PREVENTION IN NORTH VIETNAM. Int Q Community Health Educ. 2011;31: 21–31. doi: 10.2190/IQ.31.1.c 21576065 10.2190/IQ.31.1.c

[15] Davlin SL, Lapiz SM, Miranda ME, Murray KO. Knowledge, attitudes, and practices regarding rabies in Filipinos following implementation of the Bohol Rabies Prevention and Elimination Programme. Epidemiol Infect. 2013; 1–10. doi: 10.1017/S0950268813002513 24093635 10.1017/S0950268813002513 PMC9151205

[16] Ameh VO, Dzikwi AA, Umoh JU. Assessment of Knowledge, Attitude and Practice of Dog Owners to Canine Rabies in Wukari Metropolis, Taraba State Nigeria. Glob J Health Sci. 2014;6: 226–240. doi: 10.5539/gjhs.v6n5p226 25168987 10.5539/gjhs.v6n5p226 PMC4825497

[17] Digafe RT, Kifelew LG, Mechesso AF. Knowledge, attitudes and practices towards rabies: questionnaire survey in rural household heads of Gondar Zuria District, Ethiopia. BMC Res Notes. BioMed Central; 2015;8: 1–7. doi: 10.1186/s13104-015-1357-8 26328612 10.1186/s13104-015-1357-8 PMC4566865

[18] Kabeta T, Deresa B, Tigre W, Ward MP, Mor SM. Knowledge, Attitudes and Practices of Animal Bite Victims Attending an Anti-rabies Health Centre in Jimma Town, Ethiopia. PLoS Negl Trop Dis. 2015;9: 1–14. doi: 10.1371/journal.pntd.0003867 26114573 10.1371/journal.pntd.0003867 PMC4482645

[19] Tenzin T, Ahmed R, Debnath NC, Ahmed G, Yamage M. Free-Roaming Dog Population Estimation and Status of the Dog Population Management and Rabies Control Program in Dhaka City, Bangladesh. 2015; 1–14. doi: 10.1371/journal.pntd.0003784 25978406 10.1371/journal.pntd.0003784 PMC4433337

[20] Jemberu WT, Molla W, Almaw G, Alemu S. Incidence of Rabies in Humans and Domestic Animals and People ‘ s Awareness in North Gondar Zone, Ethiopia. PLoS Negl Trop Dis. 2013;7: 1–6. doi: 10.1371/journal.pntd.0002216 23675547 10.1371/journal.pntd.0002216 PMC3649954

[21] Dzikwi AA, Bello HO, Umoh JU. Impact of Rabies Education on the Knowledge of the Disease among Primary School Children in Samaru, Zaria, Nigeria. Merit Res J Educ Rev. 2015;4: 79–84.

[22] United Nations Statistics Division. No Title. In: UNData Malawi. 2015.

[23] Kahle D, Wickham H. ggmap: Spatial Visualization with ggplot2. R J. 2013;5: 144–161.

[24] Systems CR. No Title. In: https://www.surveysystem.com/sscalc.htm. 2012.

[25] Gibson AD, Handel IG, Shervell K, Roux T, Mayer D, Muyila S, et al The Vaccination of 35,000 Dogs in 20 Working Days Using Combined Static Point and Door-to-Door Methods in Blantyre, Malawi. 2016; 1–20. doi: 10.1371/journal.pntd.0004824 27414810 10.1371/journal.pntd.0004824 PMC4945057

[26] Thriemer K, Ley B, Ame SM, Puri MK, Hashim R, Chang NY, et al Replacing paper data collection forms with electronic data entry in the field: findings from a study of community-acquired bloodstream infections in Pemba, Zanzibar. BMC Res Notes. 2012; 113. 10.1186/1756-0500-5-113 PMC3392743 22353420

[27] Team Rs. R Studio: Integrated Development for R. Boston, MA: RStudio, Inc; 2015.

[28] World Health Organisation. Frequently asked questions on rabies. 2013.

[29] Leedom JM. Milk of Nonhuman Origin and Infectious Diseases in Humans. Clin Infect Dis. 2006;43: 610–615. doi: 10.1086/507035 16886155 10.1086/507035

[30] R Core Team. R: A language and environment for statistical computing. Vienna, Austria: R Foundation for Statistical Computing; 2013.

[31] Diez D, Barr C, Ҫetinkaya-Rundel M. OpenIntro Statistics, Third Edition 2015.

[32] Dean DJ, Baer GM, Thompson WR. Studies on the local treatment of rabies-infected wounds. Bull World Health Organ. 1963;28: 477–486. 14026136 PMC2554747

[33] Wilde H, Sirikawin S, Sabcharoen A, Kingnate D, Harischandra PAL, Chaiyabutr N, et al Failure of Postexposure Treatment of Rabies in Children Lakkumar Fernando, Liyanage J. B. and Sitprija Visith Published by: Oxford University Press Stable URL: http://www.jstor.org/stable/4459224 Failure of Postexposure Treatment of Rabies in Childre. 1996;22: 228–232. 10.1093/clinids/22.2.228 8838177

[34] Hampson K, Dobson A, Kaare M, Dushoff J, Magoto M, Cleaveland S. Rabies Exposures, Post-Exposure Prophylaxis and Deaths in a Region of Endemic Canine Rabies. 2008;2 doi: 10.1371/journal.pntd.0000339 19030223 10.1371/journal.pntd.0000339 PMC2582685

[35] Toyama N, Lee YM, Muto T. Japanese preschoolers’ understanding of biological concepts related to procedures for animal care. Early Child Res Q. 1997;12: 347–360. doi: 10.1016/S0885-2006(97)90008-9

[36] Miura A, Bradshawtt JWS, Tanidat H. CHILDHOOD EXPERIENCES AND ATTITUDES TOWARDS ANIMAL ISSUES: A COMPARISON OF YOUNG ADUL TS IN JAPAN AND THE UK. Anim Welf. 2002;11: 437–448.

[37] Knobel DL, Laurenson MK, Kazwala RR, Boden LA, Cleaveland S. A cross-sectional study of factors associated with dog ownership in Tanzania. BMC Vet Res. 2008;4:5: 1–10. doi: 10.1186/1746-6148-4-5 18230137 10.1186/1746-6148-4-5 PMC2262882

[38] Mbilo C, Léchenne M, Hattendorf J, Madjadinan S, Anyiam F, Zinsstag J. Rabies awareness and dog ownership among rural northern and southern Chadian communities—Analysis of a community-based, cross-sectional household survey. Acta Trop. Elsevier B.V.; 2017;175: 100–111. doi: 10.1016/j.actatropica.2016.06.003 27377767 10.1016/j.actatropica.2016.06.003

[39] Depani S, Mallewa M, Kennedy N, Molyneux E. World Rabies Day: evidence of rise in paediatric rabies cases in Malawi. Lancet. Elsevier Ltd; 2012;380: 1148 doi: 10.1016/S0140-6736(12)61668-7 10.1016/S0140-6736(12)61668-7 23021286

