# Supplementary material for: A rabies lesson improves rabies knowledge amongst primary school children in Zomba, Malawi
Source: PLoS Negl Trop Dis. 2018 Mar 9;12(3):e0006293. doi: 10.1371/journal.pntd.0006293 (PMC5862537; doi:10.1371/journal.pntd.0006293)
Supplement: S1 Appendix — (DOCX) [file pntd.0006293.s001.docx]

# S1 Appendix - Questionnaire

| Date |  | School |  |
| --- | --- | --- | --- |
| ID Code | (*a unique number given per child)* | | |

**Section A – demographics**

A1. What is your age?

| Age | 8 | 9 | 10 | 11 | 12 | 13 | 14 | 15+ |
| --- | --- | --- | --- | --- | --- | --- | --- | --- |

A2. Are you a boy or a girl?

Girl Boy

A3. What is your religion?

Christian (Catholic) Christian (other) Muslim

Other None

**Section B – Attitude towards dogs and ownership**

B1. Do you own or have regular contact with any dogs?

Yes, I own a dog Yes, I regularly have contact with a dog

No

B2. Why do you own a dog(s)? (Tick as many as you think are correct)

Guarding Hunting or Fighting Breeding

Pet/Companion Stray/Dog needed a home I don’t know

B3. Where does your dog(s) spend time? (Tick as many as you think are correct)

Inside the home Outside of the property Tied up outside your house

In a cage outside on your property Not on a chain or in a cage but in the yard

B4. Are you scared of dogs?

Yes No Sometimes

B5. Are dogs good friends for you and your family?

Yes No I don’t know

B6. Which of these dogs are safe to approach? Select one answer per row

|  | Yes - Safe | No – Not Safe | Don’t Know |  |  | Yes - Safe | No – Not Safe | Don’t Know |
| --- | --- | --- | --- | --- | --- | --- | --- | --- |
| Body Language |  |  |  |  | Tail Signals |  |  |  |
| 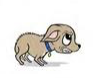 |  |  |  |  | 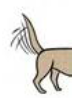 |  |  |  |
| 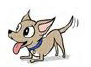 |  |  |  |  | 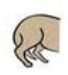 |  |  |  |
| 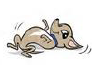 |  |  |  |  | 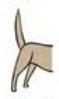 |  |  |  |
| 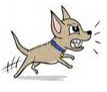 |  |  |  |  |  |  |  |  |
| 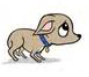 |  |  |  |  |  |  |  |  |

B7. How should you behave near a dog to avoid being bitten? (Tick as many as you think are correct)

Stand still when a dog is nearby Shout at dogs when they are nearby

Be close to the dog when they are eating Wake up the dog when it is sleeping

Wave your arms around Stare at the dog when they are nearby

Run past the dogs when they are nearby

Play and have fun with ‘happy’ dogs

Throw stones and objects at the dog

Be quiet and calm around dogs when they are nearby

Try and play with the puppies when the mother is feeding them

I don’t know Other (give examples)

B8. Do dogs have feelings?

Yes No I don’t know

B9. Does a dog need company from other dogs or people?

Yes, with other dogs Yes, with other dogs Yes, with people

Yes, with people and dogs No I don’t know

B10. How often should you give a dog food?

It should always have food Twice a day Once a day

It finds food by itself Never I don’t know

B11. How often should you give a dog water?

It should always have water Twice a day Once a day

It finds water by itself Never I don’t know

B12. If a dog looks sick do you take it to see a vet?

Yes Sometimes

No I don’t know

B13. Should dogs be on a chain?

Yes, all the time Yes, some of the time

I don’t know No

B14. Should dogs have exercise?

Yes No I don’t know

B15. What things do dogs need in their home to be happy? (Tick as many as you think are correct)

To be tied up Small area to move in To be in the sun

A blanket to lay on Big area to move in To have a shade

Something to play with Soft ground to lay on Nothing

I don’t know

**Section C – Rabies**

C1. Can people get rabies?

Yes No I don’t know

C2. Which animals get rabies? (Tick as many as you think are correct)

Dog Cat Bat

Monkey Chicken Snake

Spider Bird Mongoose

Donkey Fish I don’t know

Other: Name of animal__________________________________________________

C3. Which animals can you get rabies from? (Tick as many as you think are correct)

Dog Cat Bat

Monkey Chicken Snake

Spider Bird Mongoose

Donkey Fish I don’t know

Other: Name of animal__________________________________________________

C4. How can you get rabies from an animal? (Tick as many as you think are correct)

From the wind and air Being scratched Being bitten

From the animals fur Eating the animal From its Saliva

From touching the animals From worms From its milk

Licks on broken skin/open wounds

If the animal isn’t eating or drinking Other (name):_____________________

C5. What symptoms might a dog show if they had rabies? (Tick as many as you think are correct)

Change in bark Lots of saliva Unable to move

Staggering/problem walking Blindness Weakness

Scared of water Skin problems Biting

Increased barking Diarrhoea Death

Open mouth Cough Coma

Lumps on the animal I don’t know Other (name):

________________________________________________________________________

C6. What should you do if you are bitten by a dog? (Tick as many as you think are correct)

Inform a teacher, parent or other responsible adult

Wash the wound with soap and water for 5 minutes Rub chilli in the wound

Wash the wound with soap and water for 15 minutes Tie the dog up

Apply an antiseptic to the wound Go to the hospital

Have five anti-rabies injections Spit on the wound

Go to a traditional healer

Have a anti-rabies injection

Report the dog bite to the government veterinary laboratory

Kill the dog straight away

You don’t have to do anything I don’t know

Other (give examples)______________________________________________________

C7. How can you prevent a dog from getting rabies? (Tick as many as you think are correct)

Give the dog an anti-rabies vaccination

Give the dog an anti-rabies vaccination every year

Feed the dog special herbs Feed the dog chilli

Take the dog to a traditional healer Give the dog medicine

I don’t know Other (give examples):

________________________________________________________________________

C8. How can you stop people from getting rabies? (Tick as many as you think are correct)

Give anti-rabies vaccine to dogs

Give anti-rabies vaccine to people

Educating friends and family about rabies Being kind and caring to dogs

Sterilise dogs to stop them having puppies Remove dogs from the area

Do not spend time with animals Kill dogs in the area

I don’t know Other (state):______________________

C9. Do you think rabies is serious?

Yes No I don’t know

C10. Do you think dogs need to be vaccinated against rabies?

Yes No I don’t know

**Section D – Mission Rabies awareness & participation**

D1. Were you aware of the Mission Rabies campaign before today?

No Yes

If yes where did you hear about Mission Rabies ____________________________________

D2. Have you been taught about rabies in school?

Yes, by Mission Rabies Yes, by someone else No

D3. Have you had your dog(s) vaccinated against rabies?

Yes, by Mission Rabies Yes, by someone else No

D3ii. If yes, why did you have your dog vaccinated?

----------------------------------------------------------------------------------------------------------------

D3ii. If no, why have you not had your dog vaccinated?

----------------------------------------------------------------------------------------------------------------

D4. Have you had your dog(s) operated (neutered, castrated or spayed)?

Yes, by Mission Rabies / WVS Yes, by other No

D4ii. If yes, why did you have your dog sterilised?

----------------------------------------------------------------------------------------------------------------

D4ii. If no, why have you not had your dog sterilised?

___________________________________________________________________________

**----------------------------End of Questionnaire** -----------------------
