# Supplementary material for: A rabies lesson improves rabies knowledge amongst primary school children in Zomba, Malawi
Source: PLoS Negl Trop Dis. 2018 Mar 9;12(3):e0006293. doi: 10.1371/journal.pntd.0006293 (PMC5862537; doi:10.1371/journal.pntd.0006293)
Supplement: S1 Fig — (PDF) [file pntd.0006293.s002.pdf]

Distribution of Missing Data

Figures showing the distribution of missing data for each questionnaire type; pre, post, retention and control.

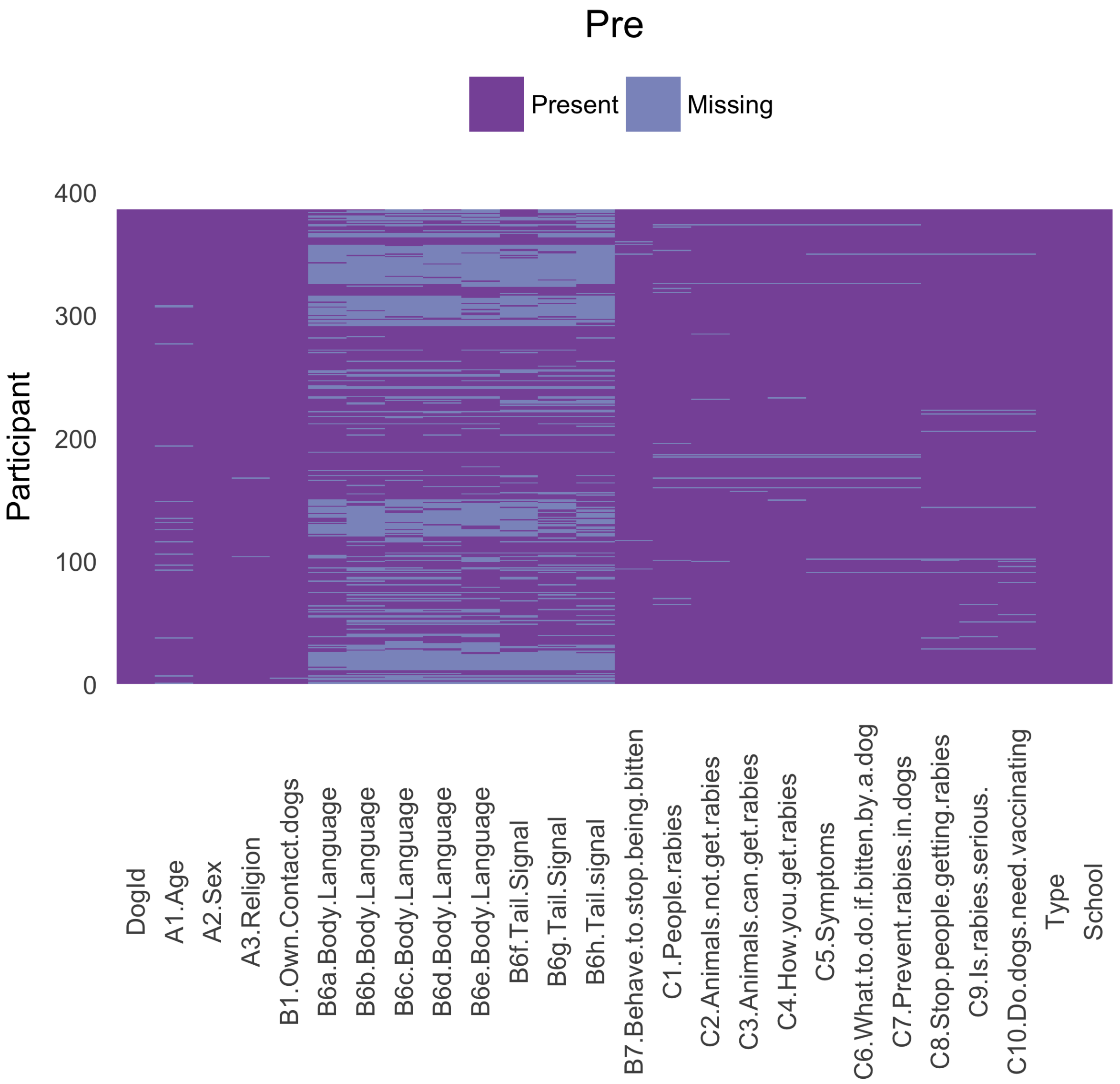

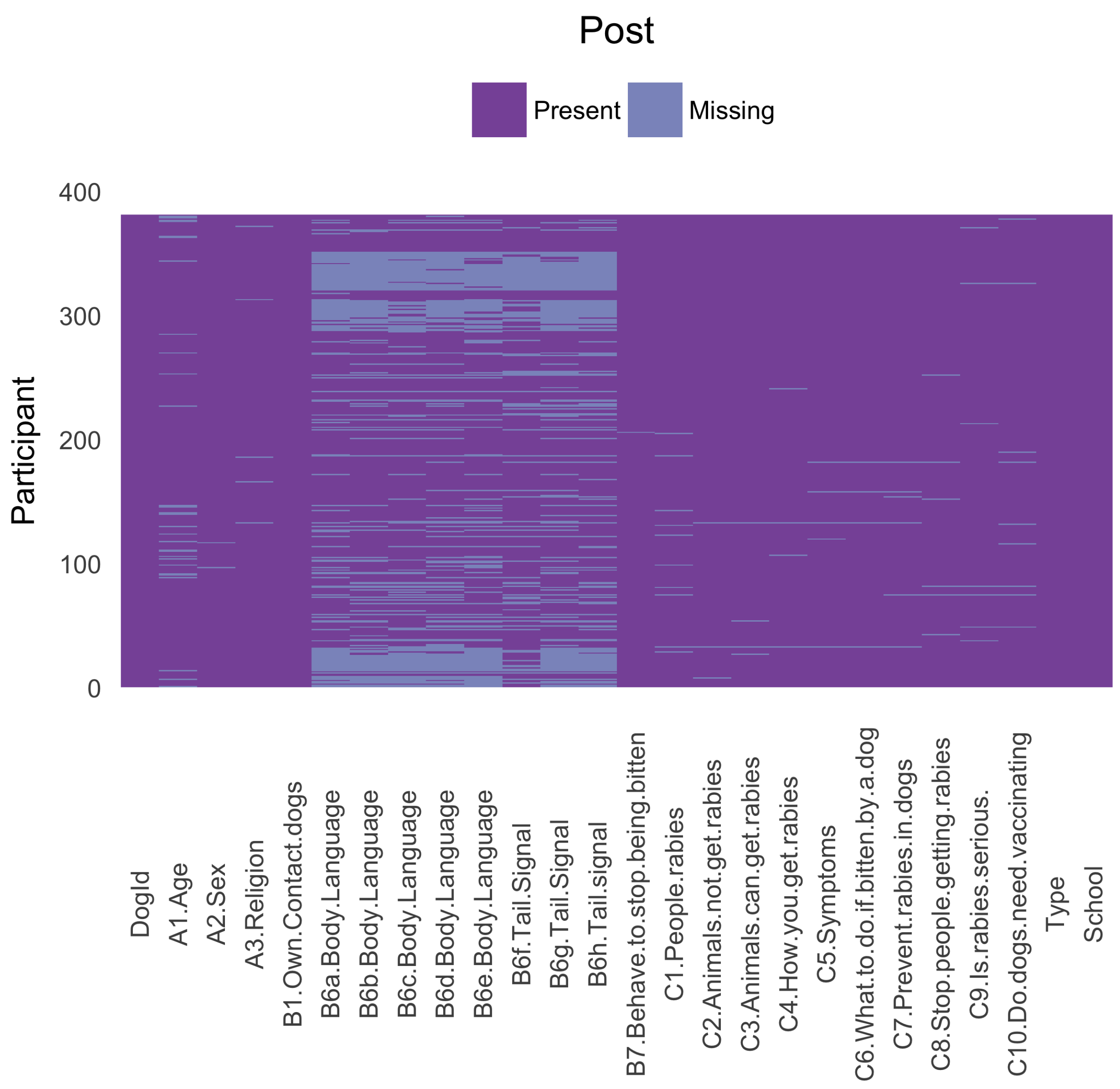
Post
Present
Missing
Participant
400
300
200
100
0
DogId
A1.Age
A2.Sex
A3.Religion
B1.Own.Contact.dogs
B6a.Body.Language
B6b.Body.Language
B6c.Body.Language
B6d.Body.Language
B6e.Body.Language
B6f.Tail.Signal
B6g.Tail.Signal
B6h.Tail.signal
B7.Behave.to.stop.being.bitten
C1.People.rabies
C2.Animals.not.get.rabies
C3.Animals.can.get.rabies
C4.How.you.get.rabies
C5.Symptoms
C6.What.to.do.if.bitten.by.a.dog
C7.Prevent.rabies.in.dogs
C8.Stop.people.getting.rabies
C9.Is.rabies.serious.
C10.Do.dogs.need.vaccinating
Type
School

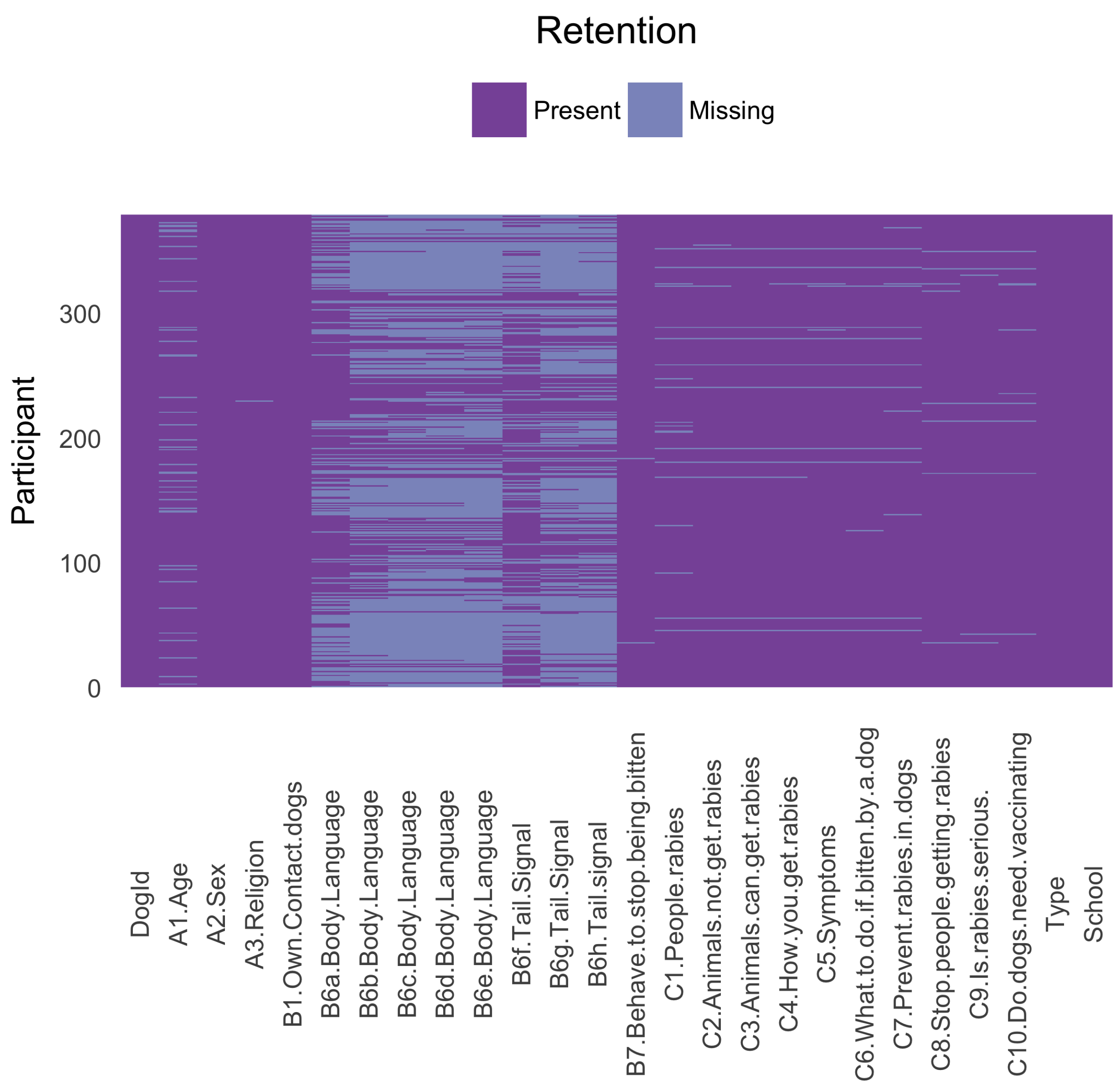
Retention
Present
Missing
Participant
0
100
200
300
DogId
A1.Age
A2.Sex
A3.Religion
B1.Own.Contact.dogs
B6a.Body.Language
B6b.Body.Language
B6c.Body.Language
B6d.Body.Language
B6e.Body.Language
B6f.Tail.Signal
B6g.Tail.Signal
B6h.Tail.signal
B7.Behave.to.stop.being.bitten
C1.People.rabies
C2.Animals.not.get.rabies
C3.Animals.can.get.rabies
C4.How.you.get.rabies
C5.Symptoms
C6.What.to.do.if.bitten.by.a.dog
C7.Prevent.rabies.in.dogs
C8.Stop.people.getting.rabies
C9.Is.rabies.serious.
C10.Do.dogs.need.vaccinating
Type
School

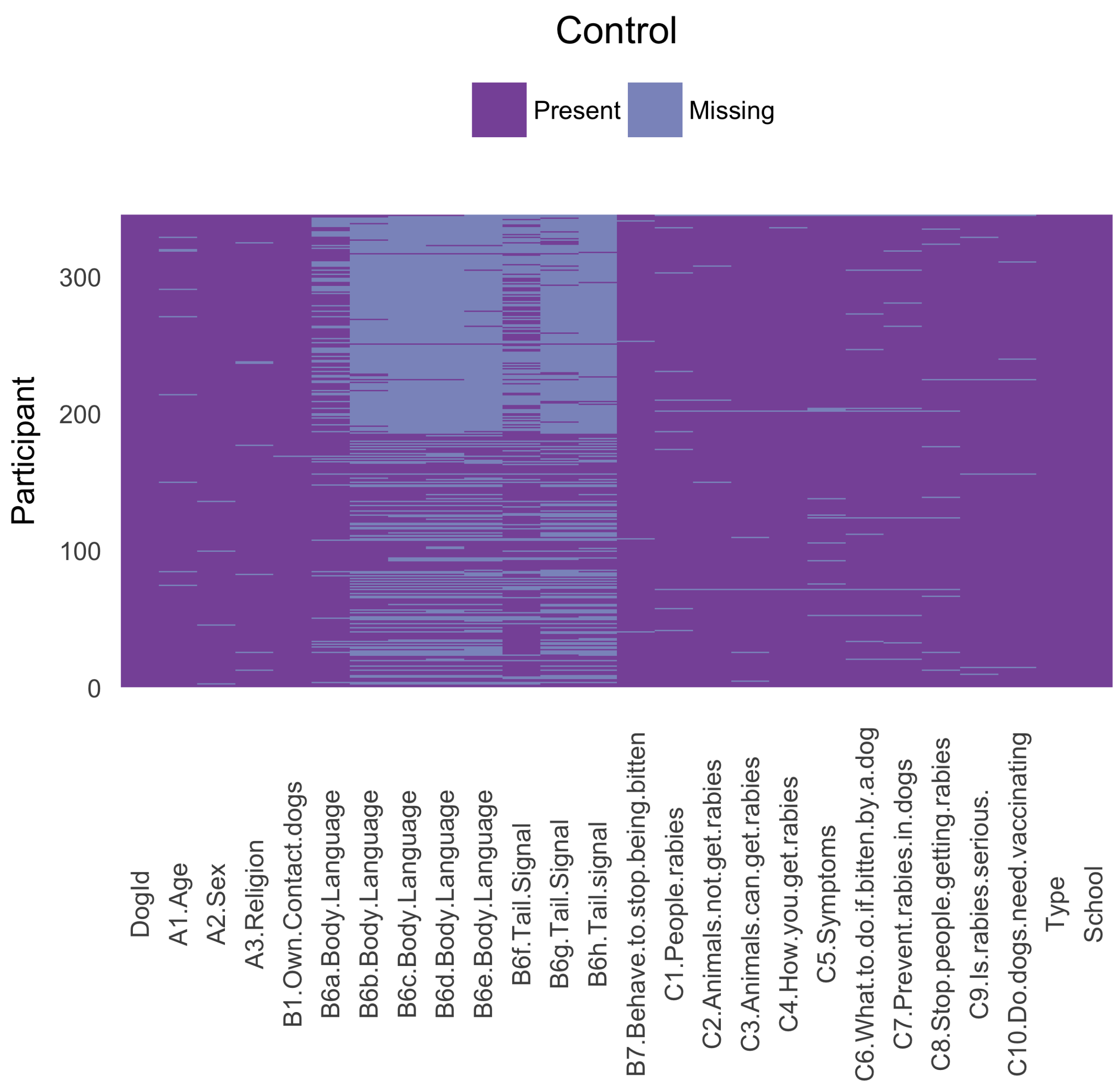

Control
Present
Missing
Participant
300
200
100
0
DogId
A1.Age
A2.Sex
A3.Religion
B1.Own.Contact.dogs
B6a.Body.Language
B6b.Body.Language
B6c.Body.Language
B6d.Body.Language
B6e.Body.Language
B6f.Tail.Signal
B6g.Tail.Signal
B6h.Tail.signal
B7.Behave.to.stop.being.bitten
C1.People.rabies
C2.Animals.not.get.rabies
C3.Animals.can.get.rabies
C4.How.you.get.rabies
C5.Symptoms
C6.What.to.do.if.bitten.by.a.dog
C7.Prevent.rabies.in.dogs
C8.Stop.people.getting.rabies
C9.Is.rabies.serious.
C10.Do.dogs.need.vaccinating
Type
School
