# Supplementary material for: A rabies lesson improves rabies knowledge amongst primary school children in Zomba, Malawi
Source: PLoS Negl Trop Dis. 2018 Mar 9;12(3):e0006293. doi: 10.1371/journal.pntd.0006293 (PMC5862537; doi:10.1371/journal.pntd.0006293)

Figures showing a comparison between study participants according to stage of intervention

The following figures show a comparison between students in the intervention schools vs. students in the control schools According to the figures below students in the two groups have a similar age, gender and dog ownership characteristics. Nevertheless, even though the proportion of muslims between the two groups is similar, the intervention group seems to have more Catholic students, while the Control group seems to have more Christians of other denominations.

Student's age

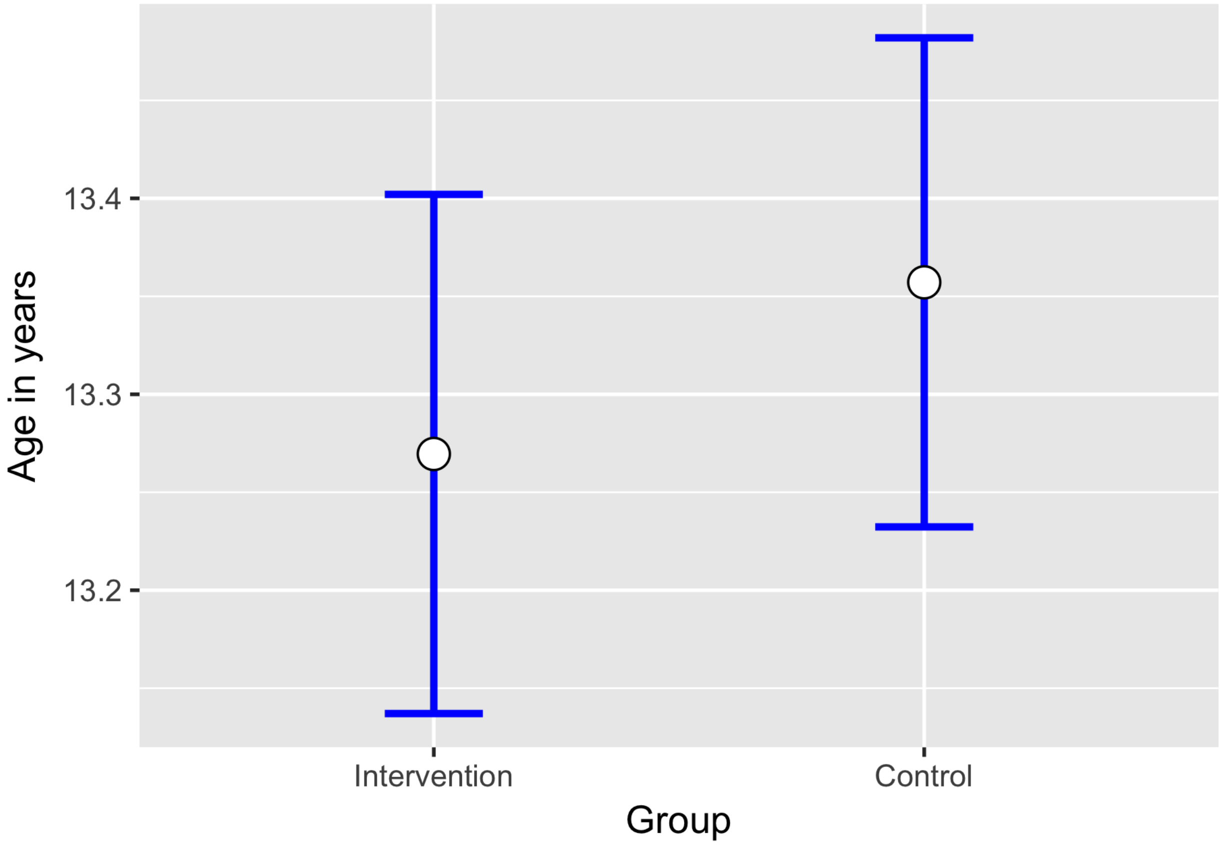

Student's gender

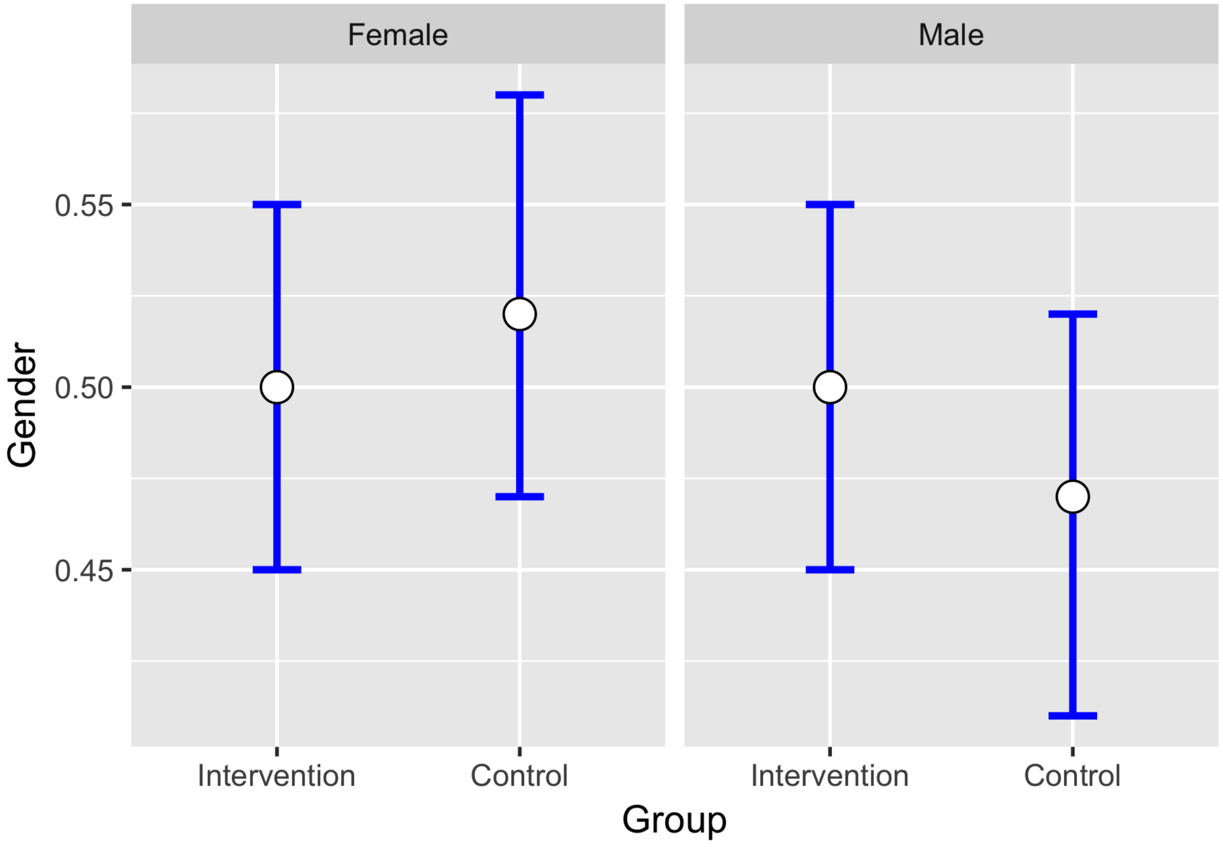

Student's religion

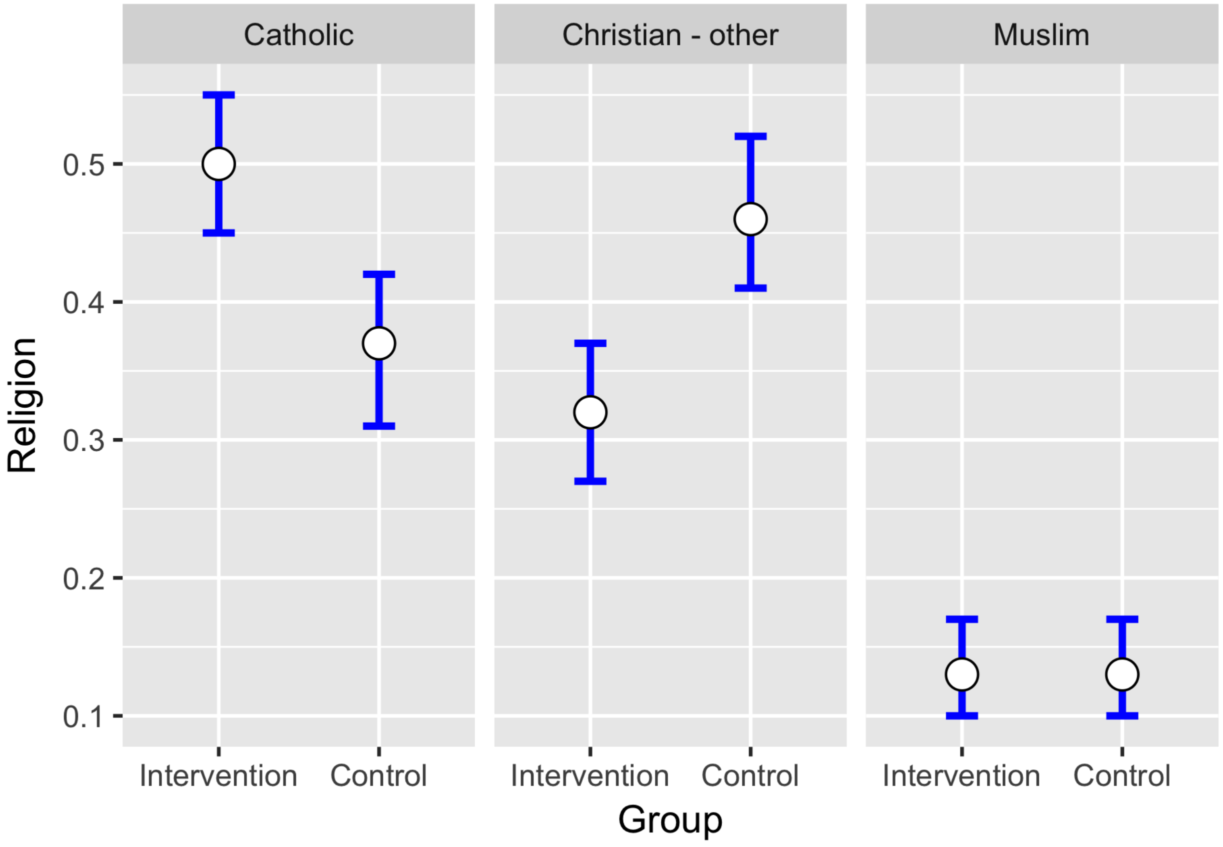

Dog ownership

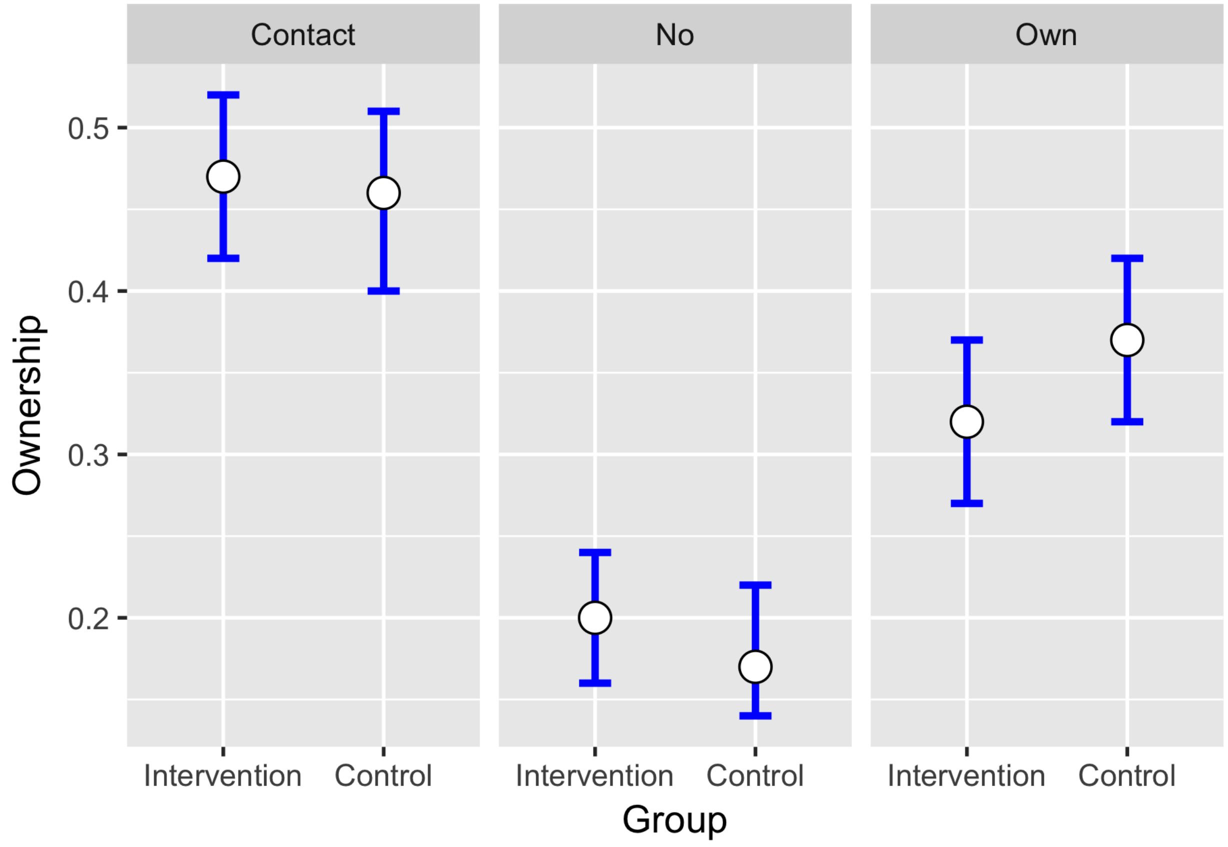

Supplement: S2 Fig — (PDF) [file pntd.0006293.s003.pdf]
