# Supplementary figures and images for: A rabies lesson improves rabies knowledge amongst primary school children in Zomba, Malawi
Source: PLoS Negl Trop Dis. 2018 Mar 9;12(3):e0006293. doi: 10.1371/journal.pntd.0006293 (PMC5862537; doi:10.1371/journal.pntd.0006293)

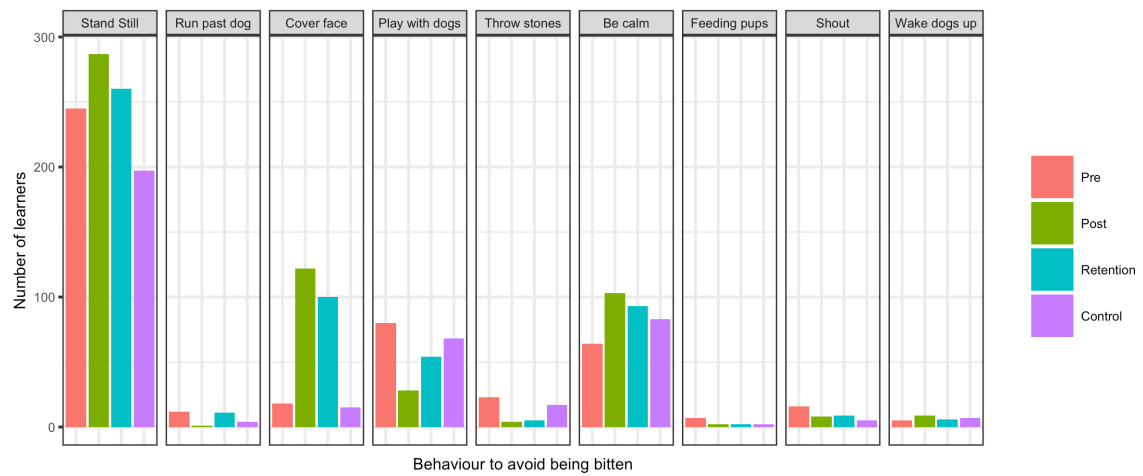

Supplement: S3 Fig — (PDF) [file pntd.0006293.s004.pdf]

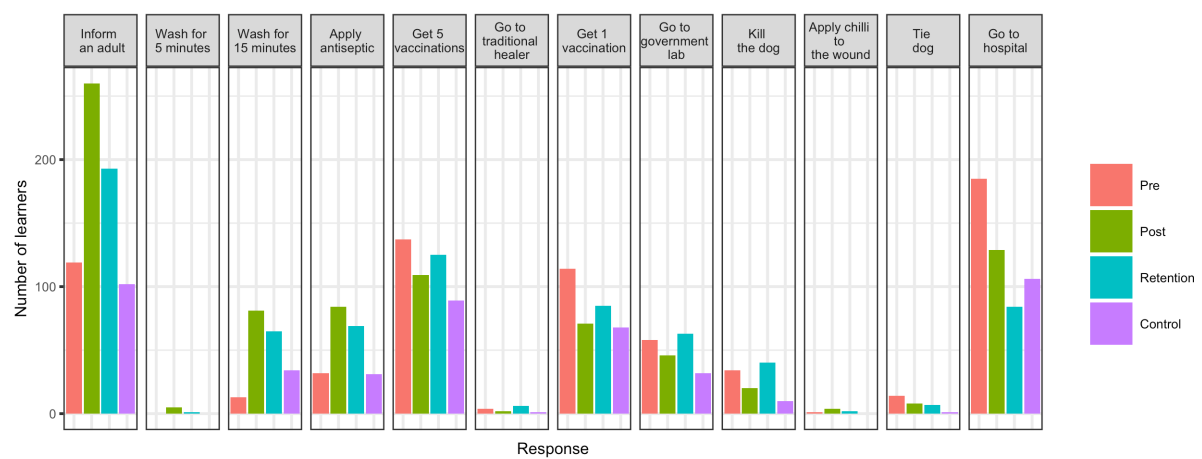

Supplement: S4 Fig — (PDF) [file pntd.0006293.s005.pdf]
