# Supplementary material for: A rabies lesson improves rabies knowledge amongst primary school children in Zomba, Malawi
Source: PLoS Negl Trop Dis. 2018 Mar 9;12(3):e0006293. doi: 10.1371/journal.pntd.0006293 (PMC5862537; doi:10.1371/journal.pntd.0006293)
Supplement: S1 Table — (DOCX) [file pntd.0006293.s006.docx]

# S1 Table. Scoring system for questionnaire responses. Each response to individual questions was allocated a numerical score to allow statistical analysis. 2 = completely correct response; 1 = correct response; 0 = wrong or no answer; -1 = very wrong answer

| **Question** | **Score given per response** | | | |
| --- | --- | --- | --- | --- |
|  | **2** | **1** | **0** | **-1** |
| C1. Can people get rabies? | Yes |  | I don't know | No |
| C2. Which animals get rabies? | Dog |  | I don't know | Chicken |
|  | Cat |  |  | Snake |
|  | Bat |  |  | Spider |
|  | Monkey |  |  | Bird |
|  | Mongoose |  |  | Fish |
|  | Donkey |  |  |  |
| C.3 Which animals can you get rabies from? | Dog |  | I don't know | Chicken |
|  | Cat |  |  | Snake |
|  | Bat |  |  | Spider |
|  | Monkey |  |  | Bird |
|  | Mongoose |  |  | Fish |
|  | Donkey |  |  |  |
| C4. How can you get rabies from an animal? | Being scratched |  | From its milk | From the wind and air |
|  | Being bitten |  | Eating the animal | From the animals fur |
|  | From its saliva |  |  | From touching the animals |
|  | Licks on broken skin/open wounds |  |  | If the animal isn't eating or drinking |
|  |  |  |  | From worms |
| C5. What symptoms might a dog show if they had rabies? | Change in bark | Staggering/problem walking | Increased barking | Blindness |
|  | Scared of water | Open mouth | Unable to move | Skin problems |
|  | Lots of saliva | Weakness | I don't know | Diarrhoea |
|  | Biting |  |  | Cough |
|  | Death |  |  | Broken neck |

| **Question** | **Score given per response** | | | |
| --- | --- | --- | --- | --- |
|  | **2** | **1** | **0** | **-1** |
| C6. What should you do if you are bitten by a dog? | Inform a teacher, parent or other responsible adult | Wash the wound with soap and water for5 minutes | Tie the dog up | Go to a traditional healer |
|  | Wash the wound with soap and water for 15 minutes | Have an anti-rabies injection#Report the dog bite to the government veterinary laboratory | I don't know | Kill the dog straight away |
|  | Apply an antiseptic to thewound |  |  | You don't have to do anything |
|  | Have 5 anti-rabies injections |  |  | Rub chilli in the wound |
|  | Go to the hospital |  |  | Spit on the wound |
| C7. How can you prevent a dog from getting rabies? | Give the dog an anti-rabies vaccination every year | Give the dog an anti-rabies vaccination | Give the dog medicine | Feed the dog special herbs |
|  |  |  | I don't know | Take the dog to a traditional healer |
|  |  |  |  | Feed the dog chilli |
| C8. How can you stop people from getting rabies? | Give anti-rabies vaccine to dogs | Sterilise dogs to stop them having puppies | Do not spend time with animals | Remove dogs from the area |
|  | Give anti-rabies vaccine to people | Being kind and caring to dogs | I don't know | Kill dogs in the area |
|  | Educating friends and family about rabies |  |  |  |
| C9. Do you think rabies is serious | Yes |  | I don't know | No |

| **Question** | **Score given per response** | | | |
| --- | --- | --- | --- | --- |
|  | **2** | **1** | **0** | **-1** |
| C10. Do you think dogs should be vaccinated against rabies? | Yes |  | I don't know | No |
