# Supplementary material for: A rabies lesson improves rabies knowledge amongst primary school children in Zomba, Malawi
Source: PLoS Negl Trop Dis. 2018 Mar 9;12(3):e0006293. doi: 10.1371/journal.pntd.0006293 (PMC5862537; doi:10.1371/journal.pntd.0006293)
Supplement: S2 Table — (DOCX) [file pntd.0006293.s007.docx]

**S2 Table. Question categorization**.

| **Category** | | **Minimum Score**  **Possible** | **Maximum Score**  **Possible** | **Question number and detail** |
| --- | --- | --- | --- | --- |
| Overall KAP | Safety around dogs | -12 | 22 | B7 How should you behave near a dog to prevent yourself from being bitten? |
|  | Rabies Knowledge | -32 | 69 | C1 Can people get rabies? |
|  |  |  |  | C2 Which animal do get rabies? |
|  |  |  |  | C3 Which animals can you get rabies from? |
|  |  |  |  | C4 How would you get rabies from an animal? |
|  |  |  |  | C5 What symptoms might a dog be showing if they had rabies? |
|  |  |  |  | C6 What should you do if you are bitten by a dog? |
|  |  |  |  | C7 How can you prevent a dog from getting rabies? |
|  |  |  |  | C8 How do you stop people from getting rabies? |
|  | Rabies Attitudes | -2 | 4 | C9 Do you think rabies is serious? |
|  |  |  |  | C10 Do you think dogs need to be vaccinated against rabies? |

Questions were grouped into categories assessing knowledge and attitudes. All responses were allocated a score and the maximum and minimum scores for each category are presented.
