# Supplementary material for: A rabies lesson improves rabies knowledge amongst primary school children in Zomba, Malawi
Source: PLoS Negl Trop Dis. 2018 Mar 9;12(3):e0006293. doi: 10.1371/journal.pntd.0006293 (PMC5862537; doi:10.1371/journal.pntd.0006293)
Supplement: S3 Table — (DOCX) [file pntd.0006293.s008.docx]

# S3 Table. Responses to rabies knowledge questions as a total number of children and as a percentage for each questionnaire category

| Rabies Knowledge | | Questionnaire | | | | | | | |
| --- | --- | --- | --- | --- | --- | --- | --- | --- | --- |
|  |  | Pre | | Post | | Retention | | Control | |
|  |  | n | % | n | % | n | % | n | % |
| Can people get rabies | |  | | | | | | | |
|  | Yes | 334 | **86.53%** | 362 | **95.01%** | 341 | **89.97%** | 300 | **86.96%** |
|  | No | 22 | **5.70%** | 6 | **1.57%** | 12 | **3.17%** | 17 | **4.93%** |
|  | I don't know | 16 | **4.15%** | 3 | **0.79%** | 6 | **1.58%** | 17 | **4.93%** |
|  | No answer | 14 | **3.63%** | 10 | **2.62%** | 20 | **5.28%** | 11 | **3.19%** |
| Animals that can get rabies | | | | | | | | | |
|  | Cat | 201 | **29.13%** | 286 | **28.37%** | 251 | **30.80%** | 219 | **34.33%** |
|  | Dog | 338 | **48.99%** | 345 | **34.23%** | 332 | **40.74%** | 309 | **48.43%** |
|  | Bat | 9 | **1.30%** | 59 | **5.85%** | 48 | **5.89%** | 20 | **3.13%** |
|  | Monkey | 57 | **8.26%** | 177 | **17.56%** | 103 | **12.64%** | 59 | **9.25%** |
|  | Chicken | 4 | **0.58%** | 10 | **0.99%** | 2 | **0.25%** | 4 | **0.63%** |
|  | Snake | 46 | **6.67%** | 32 | **3.17%** | 26 | **3.19%** | 13 | **2.04%** |
|  | Spider | 6 | **0.87%** | 6 | **0.60%** | 3 | **0.37%** | 3 | **0.47%** |
|  | Bird | 4 | **0.58%** | 7 | **0.69%** | 4 | **0.49%** | 0 | **0.00%** |
|  | Mongoose | 4 | **0.58%** | 14 | **1.39%** | 7 | **0.86%** | 8 | **1.25%** |
|  | Donkey | 19 | **2.75%** | 70 | **6.94%** | 37 | **4.54%** | 3 | **0.47%** |
|  | Fish | 2 | **0.29%** | 2 | **0.20%** | 2 | **0.25%** | 0 | **0.00%** |
| Animals that can give rabies to people | | | | | | | | | |
|  | Cat | 186 | **28.01%** | 279 | **30.13%** | 248 | **31.16%** | 199 | **31.54%** |
|  | Dog | 355 | **53.46%** | 349 | **37.69%** | 339 | **42.59%** | 324 | **51.35%** |
|  | Bat | 10 | **1.51%** | 53 | **5.72%** | 49 | **6.16%** | 23 | **3.65%** |
|  | Monkey | 48 | **7.23%** | 128 | **13.82%** | 87 | **10.93 %** | 50 | **7.92%** |
|  | Chicken | 4 | **0.60%** | 11 | **1.19%** | 4 | **0.50%** | 2 | **0.32%** |
|  | Snake | 43 | **6.48%** | 29 | **3.13%** | 24 | **3.02%** | 14 | **2.22%** |
|  | Spider | 0 | **0.00%** | 8 | **0.86%** | 4 | **0.50%** | 2 | **0.32%** |
|  | Bird | 4 | **0.60%** | 7 | **0.76%** | 4 | **0.50%** | 1 | **0.16%** |
|  | Mongoose | 3 | **0.45%** | 7 | **0.76%** | 5 | **0.63%** | 11 | **1.74%** |
|  | Donkey | 7 | **1.05%** | 48 | **5.81%** | 30 | **3.77%** | 4 | **0.63%** |
|  | Fish | 4 | **0.49%** | 7 | **0.76%** | 2 | **0.25%** | 1 | **0.16%** |
| How can people get rabies | | | | | | | | | |
|  | Wind/air | 21 | **3.93%** | 12 | **1.83%** | 32 | **4.78%** | 7 | **1.43%** |
|  | Scratch | 81 | **15.14%** | 150 | **22.83%** | 128 | **19.13%** | 84 | **17.14%** |
|  | Bitten | 318 | **59.44%** | 243 | **36.99%** | 274 | **40.96%** | 305 | **62.24%** |
|  | Animal fur | 6 | **1.12%** | 5 | **0.76%** | 16 | **2.39%** | 15 | **3.06%** |
|  | Eating animals | 17 | **3.18%** | 9 | **1.37%** | 19 | **2.84%** | 21 | **4.29%** |
|  | Saliva | 54 | **10.09%** | 190 | **28.92%** | 143 | **21.38%** | 39 | **7.96%** |
|  | Touching animals | 13 | **2.43%** | 9 | **1.37%** | 19 | **2.84%** | 5 | **1.02%** |
|  | Worms | 2 | **0.37%** | 4 | **0.61%** | 5 | **0.75%** | 1 | **0.20%** |
|  | Licking wounds | 17 | **3.18%** | 25 | **3.81%** | 27 | **4.04%** | 13 | **2.65%** |
|  | Not eating/drinking | 6 | **1.12%** | 10 | **1.52%** | 6 | **0.90%** | 0 | **0.00%** |
| Symptoms of rabies in dogs | | | | | | | | | |
|  | Change in bark | 250 | **33.07%** | 231 | **21.98%** | 225 | **22.77%** | 252 | **37.50%** |
|  | Hypersalivation | 124 | **16.40%** | 215 | **20.46%** | 180 | **18.22%** | 121 | **18.01%** |
|  | Unable to move | 12 | **1.59%** | 37 | **3.52%** | 16 | **1.62%** | 7 | **1.04%** |
|  | Staggering | 12 | **1.59%** | 44 | **4.19%** | 42 | **4.25%** | 9 | **1.34%** |
|  | Blindness | 9 | **1.19%** | 5 | **0.48%** | 13 | **1.32%** | 12 | **1.79%** |
|  | Weakness | 7 | **0.93%** | 18 | **1.71%** | 23 | **2.33%** | 4 | **0.60%** |
|  | Scared of water | 17 | **2.25%** | 164 | **15.60%** | 132 | **13.36%** | 13 | **1.93%** |
|  | Skin problems | 2 | **0.26%** | 7 | **0.67%** | 11 | **1.11%** | 4 | **0.60%** |
|  | Biting | 124 | **16.40%** | 105 | **9.99%** | 121 | **12.25%** | 126 | **18.75%** |
|  | Increased barking | 103 | **13.62%** | 89 | **8.47%** | 74 | **7.49%** | 72 | **10.71%** |
|  | Diarrhoea | 7 | **0.93%** | 5 | **0.48%** | 7 | **0.71%** | 0 | **0.00%** |
|  | Death | 11 | **1.46%** | 21 | **2.00%** | 22 | **2.23%** | 7 | **1.04%** |
|  | Open mouth | 70 | **9.26%** | 92 | **8.75%** | 84 | **8.50%** | 43 | **6.40%** |
|  | Cough | 3 | **0.40%** | 7 | **0.67%** | 16 | **1.62%** | 1 | **0.15%** |
|  | Broken neck | 3 | **0.40%** | 5 | **0.48%** | 15 | **1.52%** | 1 | **0.15%** |
|  | Lumps in skin | 2 | **0.26%** | 6 | **0.57%** | 7 | **0.71%** | 0 | **0.00%** |
| What to do if bitten | |  | | | | | | | |
|  | Inform an adult | 119 | **16.74%** | 260 | **31.75%** | 193 | **26.08%** | 102 | **21.52%** |
|  | Wash the area of 5 minutes | 0 | **0.00%** | 5 | **0.61%** | 1 | **0.14%** | 0 | **0.00%** |
|  | Wash the area for 15 minutes | 13 | **1.83%** | 81 | **9.89%** | 65 | **8.78%** | 34 | **7.17%** |
|  | Apply antiseptic | 32 | **4.50%** | 84 | **10.26%** | 69 | **9.32%** | 31 | **6.54%** |
|  | Get 5 vaccinations | 137 | **19.27%** | 109 | **13.31%** | 125 | **16.89%** | 89 | **18.78%** |
|  | Go to a traditional healer | 4 | **0.56%** | 2 | **0.24%** | 6 | **0.81%** | 1 | **0.21%** |
|  | Get 1 vaccine | 114 | **16.03%** | 71 | **8.67%** | 85 | **11.49%** | 68 | **14.35%** |
|  | Go to government lab | 58 | **8.16%** | 46 | **5.62%** | 63 | **8.51%** | 32 | **6.75%** |
|  | Kill the dog | 34 | **4.78%** | 20 | **2.44%** | 40 | **5.41%** | 10 | **2.11%** |
|  | Apply chilli to the wound | 1 | **0.14%** | 4 | **0.49%** | 2 | **0.27%** | 0 | **0.00%** |
|  | Tie the dog up | 14 | **1.97%** | 8 | **0.98%** | 7 | **0.95%** | 1 | **0.21%** |
|  | Go to the hospital | 185 | **26.02%** | 129 | **15.75%** | 84 | **11.35%** | 106 | **22.36%** |
| How to prevent rabies in dogs | | | | | | | | | |
|  | Vaccinate dogs | 171 | **37.09%** | 207 | **40.99%** | 232 | **47.35%** | 166 | **40.29%** |
|  | Vaccinate dogs annually | 249 | **54.01%** | 254 | **50.30%** | 226 | **46.12%** | 217 | **52.67%** |
|  | Feed the dog herbs | 1 | **0.22%** | 3 | **0.59%** | 5 | **1.02%** | 5 | **1.21%** |
|  | Take the dog to a traditional healer | 0 | **0.00%** | 2 | **0.40%** | 1 | **0.20%** | 7 | **1.70%** |
|  | Feed the dog chilli | 6 | **1.30%** | 6 | **1.19%** | 3 | **0.61%** | 1 | **0.24%** |
|  | Give the dog medicine | 34 | **7.38%** | 33 | **6.53%** | 23 | **4.69%** | 16 | **3.88%** |
| How to prevent people getting rabies | | | | | | | | | |
|  | Vaccinate dogs | 170 | **32.20%** | 248 | **40.39%** | 231 | **37.56%** | 163 | **36.55%** |
|  | Vaccinate people | 207 | **39.20%** | 203 | **33.06%** | 177 | **28.78%** | 163 | **36.55%** |
|  | Educate people | 78 | **14.77%** | 90 | **14.66%** | 129 | **20.98%** | 60 | **13.45%** |
|  | Sterilise dogs | 16 | **3.03%** | 18 | **2.93%** | 32 | **5.20%** | 18 | **4.04%** |
|  | Avoid animals | 34 | **6.44%** | 39 | **6.35%** | 33 | **5.37%** | 30 | **6.73%** |
|  | Remove dogs | 6 | **1.14%** | 8 | **1.30%** | 5 | **0.81%** | 3 | **0.67%** |
|  | Kill dogs | 17 | **3.22%** | 8 | **1.30%** | 8 | **1.30%** | 9 | **2.02%** |
