# Supplementary material for: A rabies lesson improves rabies knowledge amongst primary school children in Zomba, Malawi
Source: PLoS Negl Trop Dis. 2018 Mar 9;12(3):e0006293. doi: 10.1371/journal.pntd.0006293 (PMC5862537; doi:10.1371/journal.pntd.0006293)
Supplement: S1 Checklist — (DOCX) [file pntd.0006293.s009.docx]

STROBE Statement—checklist of items that should be included in reports of observational studies

|  | Item No. | Recommendation | Page  No. | Relevant text from manuscript |
| --- | --- | --- | --- | --- |
| **Title and abstract** | 1 | (*a*) Indicate the study’s design with a commonly used term in the title or the abstract | 2 | Knowledge, attitudes and practice (KAP) |
|  |  | (*b*) Provide in the abstract an informative and balanced summary of what was done and what was found | 2 | primary school children in Zomba, Malawi were given a lesson on rabies biology and preventive healthcare. Following this a mass dog vaccination programme was delivered in the same region. Knowledge, attitudes and practice (KAP) towards rabies were assessed by a questionnaire before the lesson, immediately after the lesson and 9 weeks later to assess the impact the lesson had on school children’s KAP.  Rabies knowledge and how to be safe around dogs knowledge increased following the lesson (both p<0.001), and knowledge remained higher than baseline 9 weeks after the lesson (both p<0.001). |
| Introduction | | | |  |
| Background/rationale | 2 | Explain the scientific background and rationale for the investigation being reported | 3-5 | Many people in rabies endemic countries are aware of rabies but have limited knowledge about the biology of the diseases and how rabies can be prevented (15–17). For example, in one rabies KAP study in Tanzania only 5% of those interviewed knew that they should wash a wound after a dog bite (16); over 35% of respondents in Ethiopia did not know the symptoms of rabies in people (17); and in Cambodia 52% of people that were aware of rabies knew of rabies vaccinations for dogs (7). These studies have shown that many communities lack basic knowledge about rabies, which could limit their ability to access appropriate preventive healthcare. This is a particular issue in children since most rabies KAP studies have focussed on adults despite the proportionally high incidence of rabies in children (11)  Although numerous cross sectional rabies KAP studies have been undertaken (7,15–25), few have focused on children (11,14,26). Only Dziwki *et al.* and Kanda *et al.* have evaluated the efficacy of lessons to improve rabies KAP in children (14,26). Kanda *et al.* demonstrated the effect of a 4 week rabies education programme and an educational leaflet on improving children’s KAP in Sri Lanka (14).  No published study has examined whether improvements in KAP post intervention are long lasting nor has any study evaluated efficacy of a lesson compared to a control population, which were exposed to a rabies vaccination programme but did not receive the educational intervention. |
| Objectives | 3 | State specific objectives, including any prespecified hypotheses | 5 | The aim of the study was to measure the baseline KAP of primary school children in a rabies education naive area in Zomba, Malawi, and examine how this was altered by a rabies lesson both immediately after and several weeks later. A canine rabies vaccination campaign followed the lesson and its effect on children’s KAP was investigated. |
| Methods | | | |  |
| Study design | 4 | Present key elements of study design early in the paper | 2,5 | Knowledge, attitudes and practice (KAP) towards rabies were assessed by a questionnaire before the lesson, immediately after the lesson and 9 weeks later to assess the impact the lesson had on school children’s KAP.  measure the baseline KAP of primary school children in a rabies education naive area in Zomba, Malawi, and examine how this was altered by a rabies lesson both immediately after and several weeks later. A canine rabies vaccination campaign followed the lesson and its effect on children’s KAP was investigated. |
| Setting | 5 | Describe the setting, locations, and relevant dates, including periods of recruitment, exposure, follow-up, and data collection | 6, 8 | Zomba city, a rabies education naïve area in Southern Malawi, was chosen as the study site (Fig 1)  Educated school children completed a questionnaire prior to receiving the rabies lesson (pre). Immediately following the lesson, the same school children completed the same questionnaire (post). This took place between 11^th^ and 17^th^ July 2016. The questionnaire was administered to school children in the same class at the same schools again between 7.5 and 10.5 weeks later (retention). The pre-questionnaire was used to assess school children’s baseline KAP; the post-questionnaire assessed instant impact of the lesson on school children’s KAP; and the retention questionnaire assessed longer-term learning.  The control group completed the same questionnaire only once (control), after the vaccination campaign that took place between 6^th^ and 17^th^ August 2016. This approach allowed us to assess the impact of the rabies lesson itself rather than just the exposure to the wider dog rabies vaccination programme. Retention and control questionnaires were completed between 8^th^ and 29^th^ September 2016, with retention questionnaires being conducted first and control questionnaires being conducted directly afterwards. |
| Participants | 6 | (*a*) *Cohort study*—Give the eligibility criteria, and the sources and methods of selection of participants. Describe methods of follow-up  *Case-control study*—Give the eligibility criteria, and the sources and methods of case ascertainment and control selection. Give the rationale for the choice of cases and controls  *Cross-sectional study*—Give the eligibility criteria, and the sources and methods of selection of participants | 9-10 | Standard 7 was chosen due to the number and type of questions asked, since school children in this standard were expected to be old enough to understand the questions asked and have an adequate level of literacy to read and respond to the questionnaires appropriately  school children were to be selected using a systematic random sampling method, taking every fifth student from the class register. Where the fifth student was not available the preceding learner was to be selected and so on. Due to low attendance, the proposed selection process could not be used. Instead the teacher selected 30 children to take part, or as many as were present in the class where there were less than 30 school children. The teacher was instructed to select school children at random and not to choose by ability. All school children had the opportunity to decline taking part at every stage of the study.  allocating each participant a unique identification code (UIC) consisting of a school code followed by a sequential number from 1-30 for pre/post-questionnaires; 1-60 for retention questionnaires; 1-190 for control questionnaires |
|  |  | (*b*) *Cohort study*—For matched studies, give matching criteria and number of exposed and unexposed  *Case-control study*—For matched studies, give matching criteria and the number of controls per case |  |  |
| Variables | 7 | Clearly define all outcomes, exposures, predictors, potential confounders, and effect modifiers. Give diagnostic criteria, if applicable | 13,14 | Questionnaire score was used at the outcome variable. Children’s age, gender, religion and dog ownership status were considered in the model as fixed effects. The school each student studied at was introduced in the model as a random effect. Variables selection was carried out using manual backward elimination and variables retained in the final regression model were chosen based on their effect on the Akaike information criterion (AIC). |
| Data sources/ measurement | 8* | For each variable of interest, give sources of data and details of methods of assessment (measurement). Describe comparability of assessment methods if there is more than one group | *7, 13* | The KAP of two groups of school children was evaluated and compared via a questionnaire. Children from 15 schools received the rabies lesson and were exposed to a canine rabies vaccination programme. Children from a further two schools acted as controls as they did not receive a school lesson but were exposed to the rabies vaccination programme in their catchment area. The same questionnaire was administered to all the children in the same manner in order to make the results comparable. A scoring system was developed, described below, and each questionnaire was scored in following the same scoring system. |
| Bias | 9 | Describe any efforts to address potential sources of bias | 9,10 | To minimize age related bias the questionnaire was given to standard 7 school children only  Anonymity was maintained throughout the study by allocating each participant a unique identification code (UIC) |
| Study size | 10 | Explain how the study size was arrived at | 9 | Sample size was determined using 95% confidence level, 5% margin of error and a response distribution of 50%. These parameters were chosen to give the most conservative sample size. The target population contained 2,844 standard 7 school children registered with the education department in Zomba therefore a sample size of 339 was required. This was the equivalent of 22.6 school children per school for the educated group and 169 per school for the control group. To account for incomplete questionnaires and varying school attendance this was increased to 30 per school for the educated group and 190 per school for the control group. |

Continued on next page

| Quantitative variables | 11 | Explain how quantitative variables were handled in the analyses. If applicable, describe which groupings were chosen and why | 11-13 | Numerical scores were allocated to answers based on accuracy of response for questions, which had correct or incorrect answers to allow statistical analysis. A completely correct answer scored 2, a mostly correct answer 1, a missing or wrong answer 0 and an incorrect answer -1. Scores were used as continuous variables and were not grouped into categories.  Table 1, p 12-13 |
| --- | --- | --- | --- | --- |
| Statistical methods | 12 | (*a*) Describe all statistical methods, including those used to control for confounding | 13 | Data were analysed using the statistical software R 3.3.2 (35) with paired t-tests comparing matched pre and post questionnaire responses (36). Results from 13 pre-questionnaires could not be matched to post questionnaires and this data was excluded when performing paired t-tests between these questionnaires. Two tailed two sample t-tests (36) were used to compare data that could not be matched: pre to retention, pre to control and control to retention scores. To deal with the issue of multiple testing, the threshold cut-off for significance was adjusted to a p-value < 0.003 based on the Bonferroni correction (36). Mixed effects multiple linear regression (36) was used to determine the effect of demographics on baseline questionnaire scores. Children’s age, gender, religion and dog ownership status were considered in the model as fixed effects. The school each student studied at was introduced in the model as a random effect. Variables selection was carried out using manual backward elimination and variables retained in the final regression model were chosen based on their effect on the Akaike information criterion (AIC). |
|  |  | (*b*) Describe any methods used to examine subgroups and interactions |  | NA |
|  |  | (*c*) Explain how missing data were addressed | 9,13, | Due to poor response rate to the question assessing school children’s understanding of dog body language this data was removed from analysis  Results from 13 pre-questionnaires could not be matched to post questionnaires and this data was excluded when performing paired t-tests between these questionnaires.  Data was removed from the mixed effects multiple linear regression model from any child that did not provide an answer for any of the questions used as a fixed effect in the model. |
|  |  | (*d*) *Cohort study*—If applicable, explain how loss to follow-up was addressed  *Case-control study*—If applicable, explain how matching of cases and controls was addressed  *Cross-sectional study*—If applicable, describe analytical methods taking account of sampling strategy | 10,11 | Anonymity was maintained throughout the study by allocating each participant a unique identification code (UIC) consisting of a school code followed by a sequential number from 1-30 for pre/post-questionnaires; 1-60 for retention questionnaires; 1-190 for control questionnaires. The UIC was entered on the questionnaire, a consent letter for school children’s parents/guardians and on the data entry smartphone application.  Less than half of the school children could be reliably matched between the pre and retention questionnaire due to learner absence at the time of the retention questionnaire or because school children forgot their UIC. Where there were fewer school children present for the retention than for the pre/post questionnaire, additional school children were invited to take the questionnaire with the provision that they had been present for the rabies lesson. The UIC was adapted to take this into consideration. The school code remained unchanged but the numbered portion started from 31 and ended at 60. Where the learner could remember their UIC number this was entered subsequently. |
|  |  | (*e*) Describe any sensitivity analyses | 17-18 | table 4, table 5 |
| Results | | | | |
| Participants | 13* | (a) Report numbers of individuals at each stage of study—eg numbers potentially eligible, examined for eligibility, confirmed eligible, included in the study, completing follow-up, and analysed | 14 | A total of 386 school children completed the pre-questionnaire (pre); 381 completed the post-questionnaire (post); 379 completed the retention questionnaire (retention); and 345 completed the control questionnaire (control). Only 122 learners who completed the pre-questionnaire and could remember their UIC completed the retention questionnaire. Learners who had been present for the lesson but did not complete the questionnaire and those who could not remember their UIC comprised the remainder of learners completing the retention questionnaire |
|  |  | (b) Give reasons for non-participation at each stage | 10-11 | All school children had the opportunity to decline taking part at every stage of the study.  Less than half of the school children could be reliably matched between the pre and retention questionnaire due to learner absence at the time of the retention questionnaire or because school children forgot their UIC. |
|  |  | (c) Consider use of a flow diagram |  | Fig 2 |
| Descriptive data | 14* | (a) Give characteristics of study participants (eg demographic, clinical, social) and information on exposures and potential confounders | 14-16 | The mean age of school children was 13 years old with a range between eight and 15 years old for pre, post and control groups, and nine and 15 years old for retention groups (Fig 3). Though approximately equal, slightly more females than males completed the questionnaire. (Table 2).  Approximately 50% of the educated group learners were Catholic, with other Christian denominations accounting for at least 30% of learners. This differed in the control group where more learners belonged to other Christian denominations followed by Catholicism. Across all groups between nine and 15% of learners were Muslim (Table 3). |
|  |  | (b) Indicate number of participants with missing data for each variable of interest |  | S1 Figures |
|  |  | (c) *Cohort study*—Summarise follow-up time (eg, average and total amount) |  | Fig 2 |
| Outcome data | 15* | *Cohort study*—Report numbers of outcome events or summary measures over time | *P20* | Table 6 |
|  |  | *Case-control study—*Report numbers in each exposure category, or summary measures of exposure |  |  |
|  |  | *Cross-sectional study—*Report numbers of outcome events or summary measures |  |  |
| Main results | 16 | (*a*) Give unadjusted estimates and, if applicable, confounder-adjusted estimates and their precision (eg, 95% confidence interval). Make clear which confounders were adjusted for and why they were included |  | 17 – table 4; table 5 – p 18; table 6 – p20 |
|  |  | (*b*) Report category boundaries when continuous variables were categorized | 12,17,18 | Table 1, table 4, table 5 |
|  |  | (*c*) If relevant, consider translating estimates of relative risk into absolute risk for a meaningful time period |  |  |

Continued on next page

| Other analyses | 17 | Report other analyses done—eg analyses of subgroups and interactions, and sensitivity analyses |  | Fig 8 |
| --- | --- | --- | --- | --- |
| Discussion | | | | |
| Key results | 18 | Summarise key results with reference to study objectives | 22-23 | We have shown that a short rabies lesson can significantly improve knowledge about rabies biology and preventive healthcare. This improvement in knowledge persisted at a follow up assessment over two months later. In addition, knowledge was higher than in school children who were exposed to the vaccination programme but did not receive the lesson |
| Limitations | 19 | Discuss limitations of the study, taking into account sources of potential bias or imprecision. Discuss both direction and magnitude of any potential bias | 26-27 | It was not possible to ensure that the school children who completed the pre/post questionnaire also completed the retention questionnaire preventing direct comparisons between individuals, however it was still possible to compare results as a population |
| Interpretation | 20 | Give a cautious overall interpretation of results considering objectives, limitations, multiplicity of analyses, results from similar studies, and other relevant evidence | 27 | This study assessed the impact of a rabies lesson on an education naïve population and demonstrated that this is an effective way to improve knowledge in primary school children in an urban setting. Alongside this safety around dogs also improved following a lesson. |
| Generalisability | 21 | Discuss the generalisability (external validity) of the study results | 27 | Whilst the results from this study could be extrapolated for learners in other environments, studying the effects of the lesson in different settings would provide more convincing evidence that this educational intervention is efficient in, for example, rural areas. |
| Other information | |  | | |
| Funding | 22 | Give the source of funding and the role of the funders for the present study and, if applicable, for the original study on which the present article is based |  | Not in manuscript – in funding statement as part of the submission |

*Give information separately for cases and controls in case-control studies and, if applicable, for exposed and unexposed groups in cohort and cross-sectional studies.

**Note:** An Explanation and Elaboration article discusses each checklist item and gives methodological background and published examples of transparent reporting. The STROBE checklist is best used in conjunction with this article (freely available on the Web sites of PLoS Medicine at http://www.plosmedicine.org/, Annals of Internal Medicine at http://www.annals.org/, and Epidemiology at http://www.epidem.com/). Information on the STROBE Initiative is available at www.strobe-statement.org.
